# Unearthing the fungal endophyte *Aspergillus terreus* for chemodiversity and medicinal prospects: a comprehensive review

**DOI:** 10.1186/s40694-023-00153-2

**Published:** 2023-03-25

**Authors:** Khadiga Amr, Nehal Ibrahim, Ahmed M. Elissawy, Abdel Nasser B. Singab

**Affiliations:** 1 Department of Pharmacognosy, Faculty of Pharmacy, Ain-Shams University, Organization of African Unity Street 1, Cairo, 11566 Egypt; 2 Center of Drug Discovery Research and Development, Ain-Shams University, Organization of African Unity Street 1, Cairo, 11566 Egypt

**Keywords:** *A. terreus*, Fungal endophytes, Biological activity, Natural products, Drug discovery

## Abstract

*Aspergillus terreus* microorganism represents a promising prospective source for drug discovery since it is rich in diverse kinds of bioactive secondary metabolites. It contributed to many biotechnological applications and its metabolites are used in the synthesis of certain pharmaceuticals and food products, in addition to its useful uses in fermentation processes. There are about 346 compounds identified from marine and terrestrial-derived *A. terreus* from 1987 until 2022, 172 compounds of them proved a vast array of bioactivity. This review aimed to create an up-to-date comprehensive literature data of *A. terreus’s* secondary metabolites classes supported by its different bioactivity data to be a scientific record for the next work in drug discovery.

## Introduction

Endophytes are a potential source of a wide scope of secondary metabolites possessing a sundry of biological activities opening up new scaffolds with numerous pharmaceutical, agricultural, and industrial applications [1]. There are as numerous as one million diversified fungal endophytic species existing in the inspected plants as reported approximately in 1987 by Hawksworth and Rossman [2].

Interestingly, fungi metabolize and produce a diverse array of unpretentious to very sophisticated organic compounds throughout their lifespan and most of them demonstrate some biological impacts [3]. The quantity of secondary metabolites that fungal endophytes produce is greater than that of any other class of endophytic microorganisms [4], belonging to various classes, such as steroids, terpenoids, alkaloids, isocoumarins, quinones, and phenylpropanoids in addition to lignans, phenols as well as phenolic acids, aliphatic metabolites, and lactones [4]. Indeed, the isolation of paclitaxel in 1993 from an endophytic fungus of Pacific Yew, provided a great consideration to fungal endophytes as an alternative source of bioactive secondary metabolites [5].

*Aspergillus* spp. and *Penicillium* spp. represent the most chemically examined fungal groups with hundreds of biologically active secondary metabolites [6]. *Aspergillus* is a widely distributed fungal genus that has both pathological and therapeutic impacts and it is one of the most common filamentous fungi which appertain to Ascomycetes (family Trichocomaceae), and live as endophytes, saprophytes, and parasites [2]. Antonio Micheli (1679–1736) designated this genus in his 1727 publication titled Nova Plantarum wherein 1900 plants were depicted, 1400 for the first time, among them 900 were fungi [7]. According to the World Data Center of Microorganisms (WDCM), there are approximately 378 *Aspergillus* species [2]. *A. flavus, A. fumigatus, A. niger, A. tubingensis, A. oryzae, A. versicolor, and A. terreus* are amongst the most broadly isolated and identified endophytic *Aspergillus* species [2].

A significant number of new secondary metabolites have been isolated and reported from the genus *Aspergillus*, comprising cerebroside analogues, polyketides, terpenes, sterols, alkaloids, butenolides, and peptides, and many of these compounds display fascinating biological activities [8]. The genus consists of several hundred highly aerobic mold species, which are found almost all in oxygen-rich environments and produce various beneficial extracellular enzymes and organic acids, moreover, they produce biotechnologically significant secondary metabolites [8]. A significant degree of similarity was perceived between the secondary metabolites isolated from *Aspergillus* spp. that are derived from marine and terrestrial sources with respect to their chemical skeletons and biological activities, suggesting that the metabolic systems of *Aspergillus* from marine and terrestrial sources are extremely comparable [8].

**Fig. 1:**
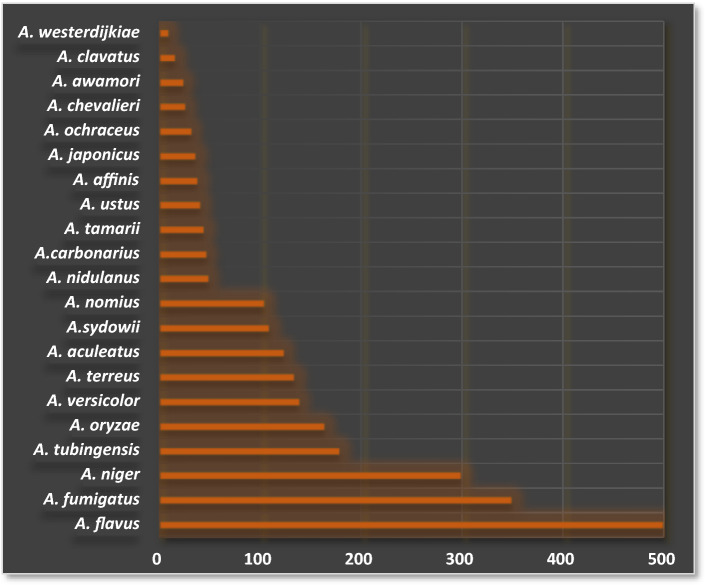
A bar chart depicting the distribution of major *Aspergillus* species, including *A. terreus,* in terms of the number of strains [9]

**Fig. 2:**
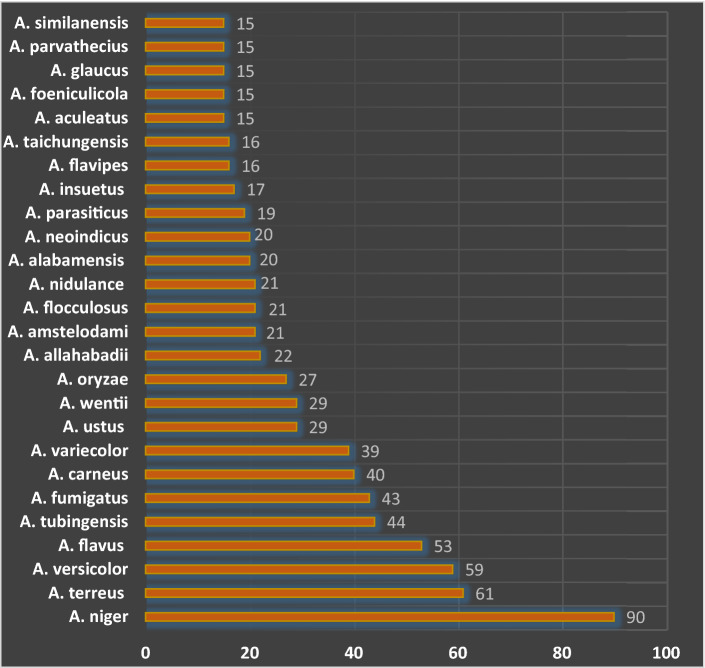
A bar chart demonstrating the number of secondary metabolites isolated from the most prevalent *Aspergillus* species [9]

Clinically, biologically, and industrially significant strains overwhelmingly come from 18 different species, as demonstrated in Fig. 1. [9]. Out of the 807 unique compounds identified following analysis of the data collected for assimilation into the *Aspergillus* Secondary Metabolites Database (*A*2MDB) for the major *Aspergillus species*, the preponderance of the secondary metabolites have been identified from approximately 25 *Aspergillus* species, *from which* *A. terreus* was among the predominant endophytes associated with various plants and the most producer of secondary metabolites as presented in Fig. 2 [2].

One of the economically significant species in the *Aspergillus* genus is *A. terreus* [10]. *A. terreus* involves in the synthesis of numerous secondary metabolites that are crucial to the food, fermentation, and pharmaceutical industries [10]. *A. terreus* is a well-known member of the genus *Aspergillus* representing the most frequently isolated species till 2019 [2]. *A. terreus* is a significant saprophytic filamentous fungus that can be found in soils [11]. *A. terreus* is an ubiquitous fungal specie in tropical and subtropical regions, nonetheless, can additionally be found in brutal environmental conditions [12], such as extreme habitats with high salt, high alkalinity, high temperature, as well as drought, and other conditions [13]. Moreover, this endophytic fungus can be isolated from different hosts, including terrestrial plants, mangrove plants, soil samples, and marine organisms as demonstrated in Table 1. The first industrial application of *A. terreus* is the production of itaconic acid, one of the most essential bioproducts in the sphere of Green Chemistry and one of the superior 12 building-block chemicals utilized in the chemical industry [11]. Furthermore, *A. terreus* isolates are utilized for the production of itatartaric acid and also for enzyme production in the fermentation industry [14].

**Table 1:** Different terrestrial and marine host sources of *A. terreus*

Strain/accession no	Host	References
FS107	Soil sample, Mauna Kea, Hawaii	[12]
MK685082	Soil sample, Penguin Island, Antarctic	[13]
NRRL 11,156	Soil sample, Tipperary, Northern Territory, Australia	[19, 37]
C-520	Soil sample, Takarazuka, Japan	[21]
HKI0499	Soil sample	[25]
23-1	Unhulled rice	[34]
NRRL 6273	Baling hay (large round or square bales)	[35]
SCSGAF0162	Tissue of the gorgonian *Echinogorgia aurantiaca*, Sanya, Hainan Province, China	[38–40]
NI	Desert soil samples (Wadi-beds, terraces and upstream, of different habitats; rocky, clay and sandy soils)	[41]
SCSIO 41008	Marine sponge *Callyspongia* sp., Xuwen, Guangdong Province, China	[41]
C9408-3	Soil of fumaroles, hot springs zone, Yangmingshan Mountain area, Taipei, Taiwan	[42, 43]
P63	Roots of the grass *Axonopus leptostachyus*	[44]
TJ403-A1	Inner part of the soft coral *Sarcophyton subviride,* Xisha Island, South China Sea	[45, 46]
LQ	Stem of rice	[47]
AH-00-51-F7	Rhizosphere of a staghorn cholla (*Opuntia versicolor* Engelm.), Sonoran Desert, Tucson Mountains, southern Arizona	[48]
NI	Rhizosphere of the canyon ragweed [*Ambrosia ambrosioides* (Cav.) Payne; Asteraceae], Sonoran Desert plant	[49]
TM8	Sub-surface soil sample, hot desert (_~_ 50 °C), South Egypt	[50, 51]
FA009	Offshore sediment, Jeju, Korea	[52]
NI	Qinghai-Tibet Plateau, east Asia	[53]
NI	Red alga *Halymenia acuminata* collected in the Bijin Island, Gyeongnam Province	[54]
IFB-E030	Stems of healthy *Artemisia annua* L. (Asteraceae), Zijin Mountain, Nanjing, China	[55, 56]
MHL-P22	Leaves of *Malus halliana*	[57]
ZDF21	Soil sample, shore sediment of Lake Koka, Ethiopia	[58]
A8-4	Mangrove-associated marine sediments, Guangxi Zhuang, Autonomous Region of China	[59]
PR-P-2	Fresh plant of *Camellia sinensis* var. a*ssamica* (Mast.) Kitam	[60, 61]
BCC 4651	Tree hole, Nam Nao National Park, Phetchabun Province, Thailand	[62, 63]
PT06-2	Sediment (saline 20%) of the Putian Sea Saltern, Fujian Province, China	[64]
GX7-3B	Mangrove *Bruguiera gymnoihiza* (Linn.) Savigny, coastal salt marsh, South China Sea, Guangxi province	[65]
SCSIO 41202	Deep-seas sediment, South China Sea coast	[66]
NI	Garbage component, organic fertilizer factory, Bangkok, Thailand	[67]
AST No. Feb 2013	Internal tissue of healthy roots of *Carthamus lanatus* L. (Asteraceae)	[68–70]
NI	Desert soil samples, Riyadh, Kingdom of Saudi Arabia	[71]
CFCC 81836	China Forestry Culture Collection Center	[72]
GX7-3B	Branch of mangrove *Brugnieria gymnoihiza* (L.) Savigny, coastal salt marsh, South China Sea, Guangxi province	[73]
H010	Mangrove plant *Kandelia obovate*	[74, 75]
QCX20130513	Soil, bottom of the Yangzi River, Wuhan	[76]
EN-539	Tissue of the marine red alga *Laurencia okamurai,* coast of Qingdao, China	[77, 78]
MK418744	Tripterygium wilfordii Hook. f. (Celastraceae)	[79]
GZU-31-1	Marine* Onchidium struma*	[80]
ML-44	Gut of Pacific Oyster	[81]
LGO13	Sediment sample from a heavy metal containing sewage water, Helwan, Egypt	[82]
Fb000501	Soil sample, Chile	[83, 84]
SC1550	Leaves of *Suriana maritima* L. (tropical plant), Yongxing Island, south China Sea, China	[85]
RCBC1002	Leaves of *Mammea siamensis,* Rayong province, Thailand	[86]
NI	Marine sponge *Phakellia fusca*	[87]
NI	Red marine alga *Laurencia ceylanica,* Arugam Bay, East coast of Sri Lanka	[88]
SCSIO FZQ028	Deep-sea sediment sample (1718 m depth), South China Sea	[89]
MXH-23	Sponge (unidentified), Naozhou Sea, Guangdong Province, China	[90]
GWQ-48	Mangrove rhizosphere soil sample, coast of Fujian province	[91]
AT20180812	Flower of *Hypericum perforatum*, Muyu, Shennongjia District,Hubei Province, People’s Republic of China	[92]
DSM 11247	Soil sample, Tamil Nadu, India	[93, 94]
13830	Soil sample, Mexico	[95, 96]
BDKU 1164	Marine sediment, Mubarak village beach, near Karachi, Pakistan	[97]
YM 39661	Stems of *Opuntia ficus-indica* Mill, Yuanjiang, Yunnan, P.R. China	[98]
QT122	Gut of healthy, mature dragonfly, Jinhua, Zhejiang, PR China	[99]
KP131622	Leaves, flowers, roots, and stem bark of *Bruguiera gymnorrhyza,* Jaffna lagoon, Northern Province, Sri Lanka	[100]
NI	Agricultural soils near to San Luis, Riobamba, Ecuador	[101]
F7	*Hyptis suaveolens* (L.) poit	[102]
MP15	Healthy old leaf of *Swietenia macrophylla* King	[103]
ENF12	Tissues of the mulberry leaf (*Morus indica* L.)	[104]
MB14-HBr	Sponge *Haliclona* species, Dongsha Atoll, Taiwan	[105]
AH1	Tissues of *Ipomoea carnea,* from polluted soil in Elbehira Governorate, Egypt	[106]

*NI* The Strain No/or Accession code was not mentioned

A wide variety of bioactive secondary metabolites have been reported from *A. terreus* isolates, that hold promise to humankind, such as lovastatin, a cholesterol-lowering drug [15], the antitumor metabolites terrein [16, 17], asterriquinone [18], and quadrone [19], antiviral compound such as acetylaranotin [20, 21], acetylcholinesterase inhibitors like territrem B (TRB) [22], in addition to butyrolactone I which holds a wide scope of biological activities as antioxidant, antidiabetic [23], antitumor [24], and antiapoptotic [25], and cyclosporine A [26]. Furthermore, mycotoxins, such as citreoviridin [27], citrinin [3, 14], cytochalasin E [28], emodin [3, 29, 30], geodin [3, 30, 31], gliotoxin [6, 32], patulin [3, 33], territrems [34], terretonin [35] as well as sulochrin were produced by *A. terreus* isolates [3].

It should be highlighted that applying a diversity of genetic and metabolic engineering approaches to the fermentation process in fungi could significantly enrich the natural compounds yield by activating silent “sleeping” gene clusters and identifying new products. It is well recognized that most biosynthetic gene clusters of fungi are silent or expressed at quite low levels under typical cultural conditions. One strain many compounds (OSMAC) strategy (changing media composition, aeration, temperature, or flask shape), interspecies crosstalk (co-culture method), and genomics-based approaches have been effectively shown to activate sleeping or cryptic biosynthetic genes (heterologous expression of orphan biosynthesis genes) [36]. The chemical-epigenetic technique, in which DNA methyltransferase inhibitors (DNMTi) or histone deacetylase inhibitors (HDACi) are used as chemical-epigenetic modifiers may successfully induce the transcription of silent biosynthetic gene clusters, resulting in the production of a diverse range of natural products with different biological potential [36].

The aim of this review was to provide a thorough survey of 346 compounds isolated from *A. terreus* from the year 1987 to the first quarter of the year 2022 and give insight into the multifaceted role of *A. terreus* as a potential source of secondary metabolites from various classes with myriad biological activities of medicinal potential. Compounds isolated from this ubiquitous filamentous fungus are categorized according to their chemical nature. Their biological significance and natural abundance from a variety of marine and terrestrial sources and habitats are discussed as well. This review can be well exploited to understand and furthermore plan for the production of promising secondary metabolites from this pervasive fungus for medicinal, industrial, and ecological applications. Sundry online resources and databases have been used through this review, including CAS (Chemical Abstract Service) search, Scifinder, Marin Lit, web of science, Springer, Elsevier, and Researchgate. Furthermore, a book chapter summarizing data on the *A. terreus* was included, along with review articles providing some data about the *Aspergillus* genus, and certain chemical classes that were covered in this review.

## Secondary metabolites isolated from *A. terreus*

The 346 secondary metabolites isolated and identified from the endophytic fungus *A. terreus* from different hosts are classified according to their chemical nature.

### Alkaloids

#### Indole alkaloids

Prenylated indole alkaloids comprise an assorted class of natural products with sophisticated chemical structures and powerful pharmacological activities [44]. Examination of the ethyl acetate (EtOAc) extract of the endophytic fungus *A. terreus* P63 obtained from roots of the *Axonopus leptostachyus*, yielded the prenylated indole alkaloid, Giluterrin (1), bearing a novel carbon skeleton [44]. Prenyl indole alkaloids are crossbred natural products biogenetically arising from amino acid and isoprenoid moieties [44]. They originate from three diverse building blocks: L-tryptophan, an acyclic amino acid residue consisting of one proline, *β*-methyl proline, or pipecolic acid, and one or two isoprene units, linked through C1 or C3 to the aromatic nucleus (regular or reverses moieties, respectively) [44]. Luteoride E (2) is a prenylated tryptophan derivative, isolated from the coral *Sarcophyton subviride-*associated fungus *A. terreus* [45]. Similarly, *A. terreus* LQ has also yielded the indole alkaloids; Chaetominine (3) and Spirotryprostatin A (4), which also represent quinazolines and spiro-indole dioxopiperazine derivatives, respectively [47].

Moreover, *A. terreus* LQ isolated from rice stem could produce sundry alkaloids with diverse structures and pharmacological activities, counting the prenylated ergot alkaloid-like compound Fumigaclavine C (5) and its structural analogue Fumigaclavine I (6). This suggests that the ergot alkaloid-like Fumigaclavine C (5) may not be biosynthetically derived from L-tryptophan and poses LQ as a prospective producer of alkaloids [47].

Tryptoquivalines are a type of indole alkaloids broadly dispersed in nature [12]. Interestingly, 24 Tryptoquivalines (A-V) have been isolated from two fungal genera. While Tryptoquivalines A-O, W and X were isolated from *Aspergillus spp*, Tryptoquivalines P–V were reported in *Neosartorya* species (*N. laciniosa, N. takakii, and N. pseudofischeri*) [12]. Tryptoquivalines A and B have been reported to display tremorgenic properties while Tryptoquivaline O exhibited antifungal activity [12].

Six indole alkaloids were isolated from *A. terreus* FS107 derived from a Hawaiian soil sample; Tryptoquivaline A (7), *N*-dehydroxy tryptoquivaline A (deoxytryptoquivaline) (8), *O*-deacetyl-tryptoquivaline A (9), Tryptoquivaline W (10), Tryptoquivaline X (11), and pyrazinoquinazoline derivative, Epifiscalin E (12) [12]. These alkaloids could be biogenetically obtained from a cyclic tripeptide-like precursor (valine-tryptophan-anthranilic acid) [12].

The cyclopentenedione, Asterredione (13) was isolated from *A. terreus* obtained from the rhizosphere of a staghorn cholla (*Opuntia versicolor* Engelm.) [48]. Furthermore, prenylated bis(indolyl) benzoquinone derivatives; Asterrelenin (14), Asterriquinone (ARQ) (15), Isoasterriquinone (16), and Asterriquinone monoacetate (ARQ monoacetate) (17) were reported [107]. Similarly, the rhizosphere fungus *A. terreus* afforded two bis-indoyl quinones namely, Asterriquinone C-1 (18) and Asterriquinone D (19), in addition to Neoasterriquinone (neoARQ) (20) [48, 107, 108].

**Fig. 3:**
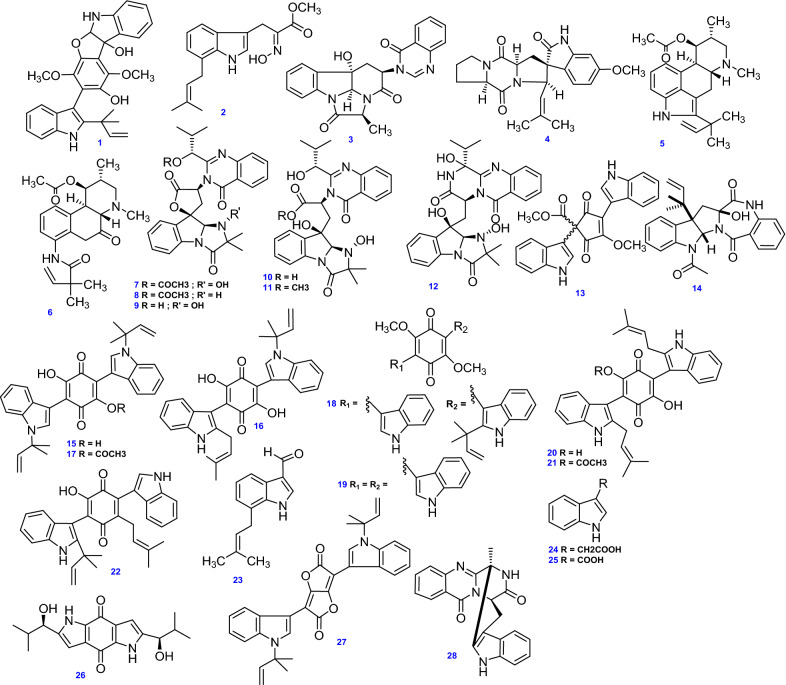
Chemical structures of indole alkaloids isolated from *A. terreus*

Additionally, two prenylated bis(indolyl) benzoquinone derivatives, Neoasterriquinone (20) and Asterriquinone SU5500 (21) were obtained from the marine-derived *A. terreus* FA009, plus, a related alkaloid Terrequinone A (22) provided by the endophytic *A. terreus* originated from the rhizosphere of the canyon ragweed [*Ambrosia ambrosioides* (Cav.) Payne; Asteraceae] [49, 52]. Three indole derivatives, 7-prenyl-indolyl-3-carbaldehyde (23), Indole-3-acetic acid (24), and Indole-3-carboxylic acid (25) were isolated from soil fungus *A. terreus* [50, 53]. A chiral dipyrrolobenzoquinone derivative; 2,6-bis[(1R)-1-hydroxyisobutyl]-1H,5Hpyrrolo[2,3-b] indole-4,8-dione (Terreusinone) (26), was obtained from the endophytic *A. terreus* isolated from the marine red alga *Halymenia acuminata* [54]. Another prenylated bis-indole alkaloid; Asterridione (ARD) (27) was also obtained from *A. terreus* IFO 6123 [107]. Moreover, ( −)-(1*R*,4*R*)-1,4-(2,3)-indomethane-1-methyl-2,4-dihydro-1*H*-pyrazino[2,1-*b*] quinazoline-3,6-dione (28) was isolated under high salinity medium (10% salt) from marine-derived *A. terreus* PTO6-2 [64]. All 28 indole alkaloids (1–28) isolated and identified from *A. terreus* are depicted in Fig. 3.

#### Ardeemins and cytochalasins alkaloids

**Fig. 4:**
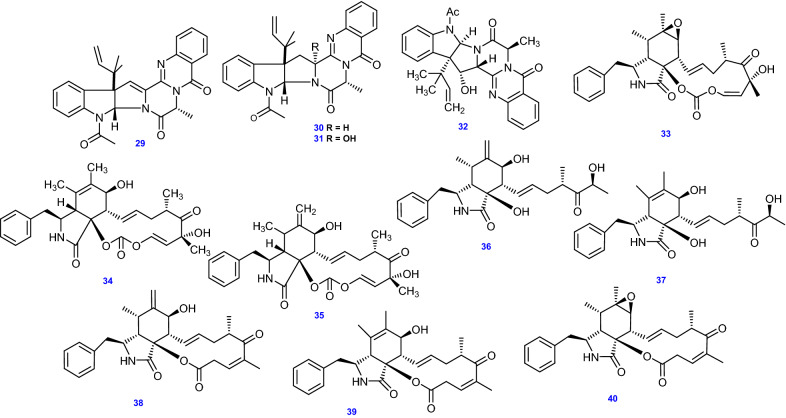
Chemical structures of Ardeemins and Cytochalasins isolated from *A. terreus*

On the investigation of *A. terreus* strain IFB-E030 inhabiting the stem of a healthy *Artemisia annua*, four ardeemins compounds have been isolated, 15b-dehydro-5-*N*-acetylardeemin (29), 5-*N*-acetylardeemin (30), 15b-*β*-hydroxy-5-*N*-acetylardeemin (31), and 16-*α*-hydroxy-5*N*-acetylardeemin (32), together with eight cytochalasins, Cytochalasin E (33), 5,6-dehydro-7-hydroxy cytochalasin E (34) and its *∆*^6,12^—isomer (35), Cytochalasin Z11 (36), Cytochalasin Z13 (37), 10-phenyl-(12)-cytochalasin Z16 (38),10-phenyl-(12)-cytochalasin Z17 (39), and Rosellichalasin (40) [55, 56]. All 12 ardeemins and cytochalasins alkaloids (29–40) isolated and identified from *A. terreus* are depicted in Fig. 4.

#### Diketopiperazines (DKPs)/dioxopiperazines/piperazinediones

**Fig. 5:**
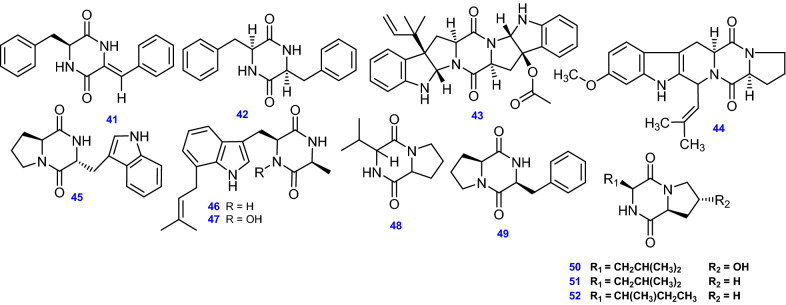
Chemical structures of DKPs isolated from *A. terreus*

Diketopiperazines (DKPs)/dioxopiperazines/piperazinediones are known as an interesting class that is numerous in a diversity of natural resources [109]. The 2,5-DKPs exist in a wide range of natural products, and this subunit is frequently seen on its own or inserted in larger and more sophisticated chemical structures from fungi, bacteria, the plant kingdom, and mammals [109]. Because of their capability to adhere to a broad scope of receptors, these compounds exhibit a wide spectrum of biological activity, rendering them enticing platforms for the exploration of drugs [109]. For instance, the endophytic fungus *A. terreus* MHL-P22 inhabiting the fresh leaves of *Malus halliana* produced (3*S*,6*Z*)-3-benzyl-6-benzyliden 2,5-dioxopiperazine (41), and (3*S*,6*S*)-3,6-dibenzyl-2,5-dioxopiperazine (42) [57]. Amauromine B (43), Fumitremorgin C (44), and Brevianamide F (45) were produced by the endophytic fungus *A. terreus* derived from different hosts, holding an indole containing DKP moiety [41, 58, 110]. Similarly, the compounds Terezine D (46) and 14-hydroxyterezine D (47), also showed indole moiety [53]. Furthermore, Cyclo (Val-Pro) (48), Cyclo- (L -Pro-L –Phe) (49), Cyclo-[L-(4-hydroxyprolinyl)-L-leucine] (50), Cyclo (Leu-Pro) (51), and Cyclo (Ile-Pro) (52) have been reported from different strains of *A. terreus* [53, 59]**.** All 12 DKPs (41- 52) from *A. terreus* are depicted in Fig. 5.

#### Epipolythiodiketopiperazines (epipolythiodioxopiperazine)(ETPs)

Epipolythiodiketopiperazines (ETPs) represent an unusual class of fungal metabolites derived from diketopiperazines (DKPs) distinguished by two or more sulfide bonds, mostly displaying powerful biological activity [60]. Since the discovery of gliotoxin in 1936, about twenty families have been described of ETPs [60], particularly, epidithiodiketopiperazines which in turn include aranotins, hyalodendrins, gliotoxins, emestrins, epicorazines, and emethallicins, were isolated from many genera; *Aspergillus, Penicillium, Hyalodendron, Emericella*, *Podospora* and *Epicoccum* [60].

**Fig. 6:**
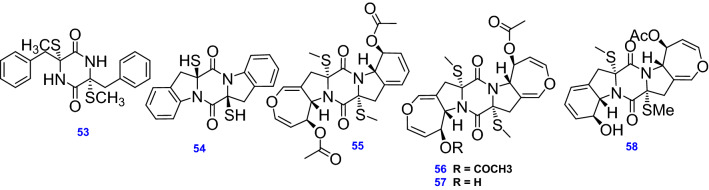
Chemical structures of ETPs isolated from *A. terreus*

The reported compounds produced by *A. terreus* from this distinctive class are (3R,6R)-3,6-dibenzyl-3,6-bis(methylthio)-2,5-dioxopiperazine (53), Asperterzine (54) a symmetric aromatized derivative of ETP [57], along with its aranotin -type diketopiperazine analogs; Bisdethiobis(methylthio)-acetylapoaranotin (55) [57], and Bisdethiobis(methylthio)-acetylaranotin (56) [60], Bisdethiobis(methylsulfanyl)aranotin (Alternarosin A) (57) and Bisdethiobis(methylsulfanyl)apoaranotin (58) [62]. All 6 ETPs (53–58) isolated and identified from *A. terreus* are depicted in Fig. 6**.**

#### Other alkaloids and nitrogenous compounds

**Fig. 7:**
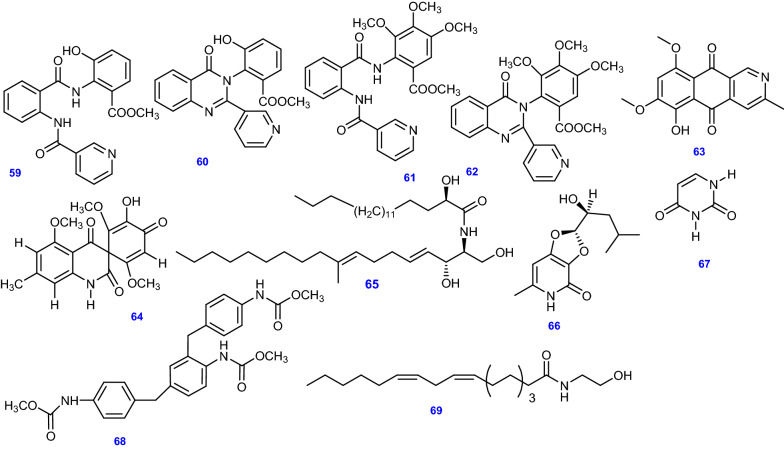
Chemical structures of other alkaloids and nitrogenous compounds isolated from *A. terreus*

Five pyridine alkaloids, Terremide A & B (59& 60), Preterremide C (61) [64], Sterremide C (62) [38], and a pyridine-containing polyketide compound, 8-*O*-methylbostrycoidin (63) were obtained from marine-derived *A. terreus* [65]. In addition, Asperfumoid (64), a spiro-quinoline alkaloid, was isolated from endophytic *A. terreus* LQ obtained from the stem of rice [47]. Other nitrogenous compounds were isolated from *A. terreus* including the ceramide, Lactariamide B (65), and the pyridine-containing compound, Dihydroisoflavipucine (66) [53], Uracil (67) [50], the peculiar *N*-phenyl-carbamic acid methylester trimer, Asperteramide A (68) [46], and the bioactive fatty acid derivative, (9*Z*,12*Z*)-*N*-(2-hydroxyethyl) octadeca-9,12-dienamide (69) [66]. All 11 other alkaloids and nitrogenous compounds (59–69) isolated and identified from *A. terreus* are depicted in Fig. 7.

### Peptides

A variety of peptides have been reported in *A. terreus* including the trimeric cyclo-depsipeptide, Beauvericin (70) [65], the cyclic tetrapeptides, Asperterrestide A & B (71 & 72) along with ($-$)-Serantrypinone (73) and ($-$)-Alantrypinone (74) [38].

Aspergillamides, a sort of modified tripeptides with unique dehydrotrytamine moieties, were basically isolated from the marine-derived *Aspergillus* fungi with unpretentious cytotoxicities, exhibiting structural variability emerging from geometric isomerization of double bonds and different categories of amino acids [41]. Around ten natural aspergillamides have been revealed [41]. Aspergillamide A, its isomer Aspergillamide B (75 & 76, respectively), Aspergillamide C, its isomer Aspergillamide D (77 & 78, respectively), Cis-L-Phenylalaninamide and Trans-L-Phenylalaninamide (79 & 80, respectively) were obtained from *A. terreus* SCSIO 41008 residing in the marine sponge *Callyspongia* sp. [41].

The cycloheptanetriones, Terretriones A–C (81–83) were biosynthesized by the endophytic fungus *A. terreus* most probably by the condensation of amino acids containing hydrophobic side chain (leucine, valine, and isoleucine, respectively) with phenylalanine analogue residues [59]. Unlike diketopiperazines, an extra carbonyl group was arbitrarily embedded between amino-nitrogen and α-carbon of phenylalanine. The cyclization reactions create cycloheptanetrione seldom found in microbial metabolites [59].

The lumazine peptide Terrelumamide A (84) was obtained from the thermophilic fungus *A. terreus* TM8 [50], while Terrelumamide B (85) was obtained from the fungal strain *A. terreus* FA009 which was derived from marine sediments. Additionally,* A. terreus* isolate from a garbage component yielded Penilumamide E (86) [67], whereas the flavin Lumichrome (87) was isolated from the endophytic *A. terreus* LQ obtained from the stem of rice [47].

**Fig. 8:**
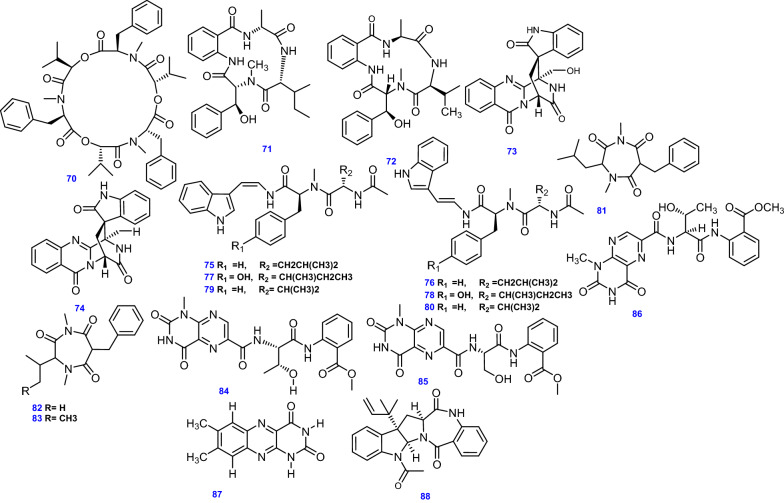
Chemical structures of peptides obtained from *A. terreus*

Interestingly, a benzodiazepine fungal metabolite, *epi*-Aszonalenin A (88) that was initially reported in *Aspergillus novofumigatus* [111], was isolated from *A. terreus* obtained from a garbage component at an organic fertilizer factory [67]. All 19 peptides (70–88) isolated and identified from *A. terreus* are depicted in Fig. 8.

### Sterols and triterpenes

The stigmasterol derivatives; (22*E*, 24*R*)-stigmasta-5,7,22-trien-3-β-ol (89), Stigmast-4-ene-3-one (90), and Stigmasta-4,6,8(14), 22-tetraen-3-one (91), have been isolated from the endophytic fungus *A. terreus* obtained from the roots of *Carthamus lanatus* [68]. In addition, the phytosterol derivative, Glucopyranosyl-*β-*sitosterol (92) was obtained from red marine alga *Laurencia ceylanica*, J. Agardh [88].

**Fig. 9:**
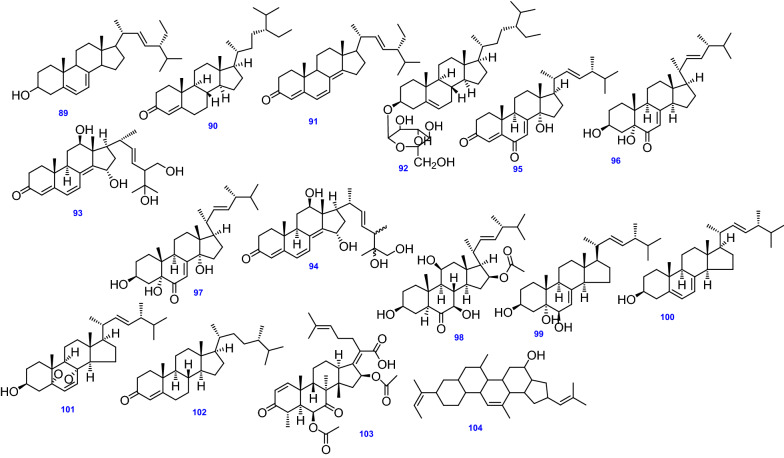
Chemical structures of sterols and triterpenes isolated from *A. terreus*

Likewise, two ergostane derivatives namely, 12*β*,15*α*,25,26-tetrahydroxyergosta-4,6,8(14),22-tetraen-3-one (94), and 12*β*,15*α*,25,28-tetrahydroxyergosta-4,6,8(14),22-tetraen-3-one (93) were obtained from the endophytic fungus *A. terreus* BCC4651 [62]. Other ergostane derivatives obtained from *A. terreus* include 14*α*-hydroxyergosta-4,7,22-triene-3,6-dione (95) [45], 3*β*,5*α*-dihydroxy-(22*E*,24*R*)-ergosta-7,22-dien-6-one (96), 3*β*,5*α*,14*α*-trihydroxy-(22E,24R)-ergosta-7,22-dien-6-one (97), NGA0187 (98) [65], (3β,5α,6β)-3,5,6-trihydroxy-ergosta-7,22-diene (99), Ergosterol (100) [50], Ergosterol peroxide (101) [110], Ergost-4-ene-3-one (102) [82]. The antibacterial nortriterpenoid, Helvolic acid (103) [112, 113] and the terpenoidal compound, Amhezole (104) were separated from soil samples [71]. All 16 Sterols and Triterpenes (89–104) isolated and identified from *A. terreus* are depicted in Fig. 9.

### Sesquiterpenes

The endophytic fungus *A. terreus* isolated from deep-sea sediment collected from the YapTrench at a depth of 4159 m yielded the carotane-type sesqiterpene Aspterric acid (105), along with two biogenetically related farnesol derivatives, Aspterric A (106) and Aspterric B (107) [114].

A series of oxygenated brasilane-type sesquiterpenoids bearing an α, β-unsaturated ketone unit named Brasilanones A-F (108–113) were identified from the endophytic fungus *A. terreus* (No. CFCC81836) [72]. The peculiar skeleton of brasilane-type sesquiterpenoids sparked considerable attention because of their different sesquiterpene skeleton comprising five methyl groups and a 5/6 bicyclic carbon skeleton [72]. As far as authors perceived, just 25 naturally occurring brasilane sesquiterpenoids have been identified, and most of them were derived from sea hare, alga, and liverwort. However, brasilane sesquiterpenoids have recently been discovered as well from basidiomycetes and endophyte fungi [72].

Conjointly, four sesquiterpenes including, Botryosphaerin B (114), Botryosphaerin F (115), 13,14,15,16-tetranorlabd-7-ene-19,6b:12,17-diolide (116), and LL-Z1271β (117), were isolated from the mangrove *Brugnieria gymnoihiza* (L.) Savigny derived *A. terreus* [73]. Furthermore, (-)-γ-Cadinene (118) and Aristolochene (119), were isolated from seed cultures of *A. terreus* NRRL ll, 156 [37].

**Fig. 10:**
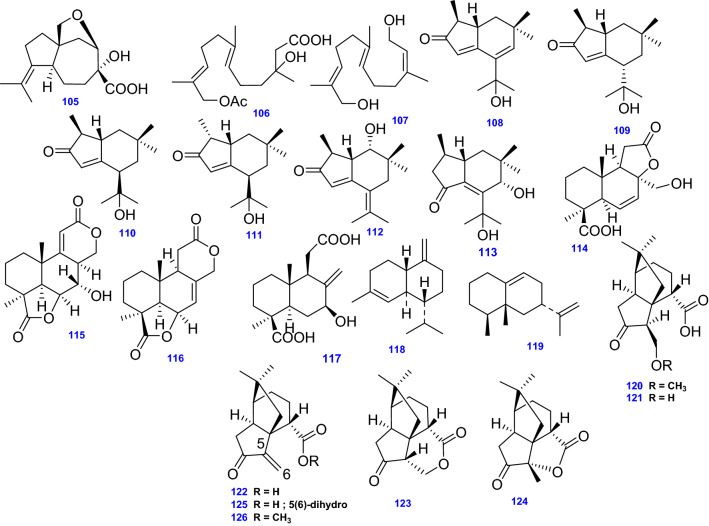
Chemical structures of sesquiterpenes isolated from *A. terreus*

*A terreus* Thom derived from the rhizosphere of a staghorn cholla (*Opuntia versicolor* Engelm.) afforded seven sesquiterpene derivatives; ( +)-5(6)-dihydro-6-methoxyterrecyclic acid A (120), ( +)-5(6)-dihydro-6-hydroxyterrecyclic acid A (121), ( +)-Terrecyclic acid A (122), (-)-Quadrone (123), (-)-Isoquadrone (124), 5(6)-dihydro-terrecyclic acid A (125), and ( +)-Terrecyclic acid A methyl ester(126) [48]. All 22 Sesquiterpenes (105–126) isolated and identified from *A. terreus* are depicted in Fig. 10.

### Sesterterpenes

**Fig. 11:**
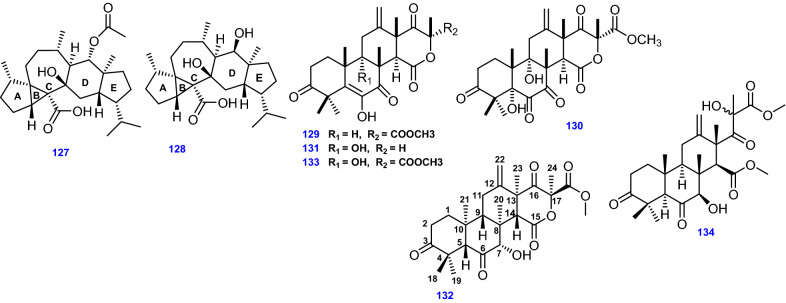
Chemical structures of sesterterpenes isolated from *A. terreus*

Sesterterpenoids are a comparatively less occurring natural members of terpenoids found in insects, fungi, lichens, terrestrial plants as well as marine organisms and a few of them have been identified from the genus *Aspergillus* [115]. Aspterpenacids A and B (127 & 128) are two sesterterpenoids possessing unique carbon skeleton of a 5/3/7/6/5 ring system that were isolated from the endophytic fungus *A. terreus* H010 obtained from the mangrove plant *Kandelia obovata* [74] as illustrated in Fig. 11.

Other six sesterterpenes reported in *A. terreus* include: Terretonin A (129), Terretonin B (130), Terretonin C (131), Terretonin D (132) and Terretonin (133) [115]. Besides, Terretonin G (134) was obtained from *A. terreus* EN-539 isolated from the marine red alga *Laurencia okamurai* [77] as compiled in Fig. 11.

### Meroterpenoids

Meroterpenoids are a hallmark class of fungal metabolites that integrate polyketide-terpenoid structures [42]. The 3,5-Dimethylorsellinic acid-based (DMOA-based) meroterpenoids are a sophisticated family of fungal secondary metabolites featuring intricate and captivating skeletons generated from a basic aromatic tetraketide 3,5-dimethylorsellinic acid and mostly isolated from *Aspergillus* and *Penicillium* species [116]. About of 100 compounds have been depicted as members of this family, after the first isolation of a 3,5-dimethylorsellinic acid-based meroterpenoid in 1976 [117]. Being versatile in pharmacological activities and novel structures, this family has drawn significant consideration [79].

Ten meroterpenoids, Asperterpenes D − M (135–144) were isolated from soil-derived *A. terreus*, and the NMR data of (135) is close to that of Terretonin (133), a DMOA-based meroterpenoid previously isolated from *A. terreus* with 11 degrees of unsaturation. The key distinction between (135) and (133) was the bearing of an oxygenated methine, the absence of methylene, and the additional degree of unsaturation in (135) which indicate a 9,11-epoxy ring in compound (135) [76].The planar structure of compound (136) was the equivalent of that of Terretonin D (132) [76]. The NOESY spectrum revealed the relative configurations of H-7, H-9, and H-14 of compound (136) β-oriented, like those of (132) [76]. Besides, the NOESY correlation between H-5 and Me-20 demonstrated that H-5 is α-oriented, which recommended compound (136) is the C-5 epimer of (132) [76].

Three unique austinoid meroterpenoids possessing fascinating spiro-lactone scaffolds: 1,2-dehydro-terredehydroaustin (145), Acetoxydehdroaustin B (146), as well as 1,2-dehydro-acetoxydehydroaustin B (147), were isolated from the mangrove *Kandelia obovata* endophytic fungus *A. terreus* H010 [75]. Most of the previously identified austinoid moreterpenoids were dextro-rotatory with positive optical rotation properties, compounds (145–147), represent the infrequently detected levo-rotatory austinoids obtained for the first time from *A. terreus* [75].

Two meroterpenoids; Yaminterritrem A (148) and Yaminterritrem B (149) were isolated by Liaw et al., from *A. terreus* from hot spring zones in Taiwan [42]. Moreover, endophytic *A. terreus* associated with the root of *Tripterygium wilfordii* Hook. f. (Celastraceae) afforded six spiro-dioxolane-containing adducts possessing 3,5-DMOA-based meroterpenoid and 2,3-butanediol moieties, Spiroterreusnoids A–F (150–155) [79]. Interestingly, a highly selective acetylcholinesterase (AChE) inhibitor, Arisugacin A (156), originally isolated from *Penicillium* sp. FO-4259 was isolated from *A. terreus* [39, 118], along with Arisugacin D (157) [40], and Arisugacin H (158) [39]. Additionally, Aspermeroterpenes A-C (159 – 161) were obtained by Tang et al., from the marine-derived *A. terreus* GZU-31-1 isolated from *Onchidium struma,* and reportedly, compound (159) possessed an extraordinarily engorged 5/3/6/6/6/5 hexacyclic skeleton [80]. Aperterpenes N–O (162–163) were derived from endophytic *A. terreus* EN-539 associated with red alga *Laurencia okamurai* [77]. Eleven highly oxygenated meroterpenoids, Terreustoxins A−K (164–174) were obtained from soil-derived *A. terreus*, however, Terreustoxins A−D (164–167) are unusual terretonins comprising two ortho-hydroxy groups at C-6 and C-7 [13]. Austalides B, N, and O (175–177) were derived from *A. terreus* 3.05358 [110]. Additionally, Territrem A (178) was derived from *A. terreus* after isolation from the coral *Sarcophyton subviride* [45]. Likewise, merosesquiterpene containing a phenyl α-pyrone (territrem derivative) Territrem B (179) was derived from the endophytic fungus *A. terreus* obtained from the sea sediment [64].

Other territrem derivatives were obtained from the endophytic fungus *A. terreus* SCSGAF0162 derived from the coral *Echinogorgia aurantiaca*, namely, Territrem C (180), Territrem D (181), Territrem E (182), 11a-dehydroxyisoterreulactone A (183), [39]. On the other hand, Terrenoid (184) [13], with a highly oxygenated tetracyclic skeleton, Terretonin D1 (185) [81], Terretonin J (186) [13], and Terretonin M (187) [50], were obtained from different strains of the endophytic fungus *A. terreus*. Similarly, a highly oxygenated tetracyclic meroterpenoid Terretonins N (188) was derived from the extremophilic *A. terreus* LGO13, while Terretonin O (189) was isolated separately from both thermophilic *A. terreus* TM8 and marine *A. terreus* LGO13 [82].

Unusual microbial meroterpenoids, Terreulactones A, B, C, and D (190–193) were isolated from *A. terreus* Fb000501 [83]. Terreulactone A is a sesquiterpene lactone-type meroterpenoid consolidating a remarkably combined lactone skeleton in its sesquiterpene moiety [83, 84]. Isoterreulactone A (194) was also isolated from *A. terreus* Fb000501 [119].

The 3,5-DMOA-based meroterpenoids, Terreusterpenes A–C (195–197), were identified in *A. terreus* isolated from the inner part of the soft coral *Sarcophyton subviride* [120]. Terreusterpenes A and B typify a unique group of meroterpenoids comprising 2,3,5-trimethyl-4-oxo-5-carboxy tetrahydrofuran moiety [120].

**Fig. 12:**
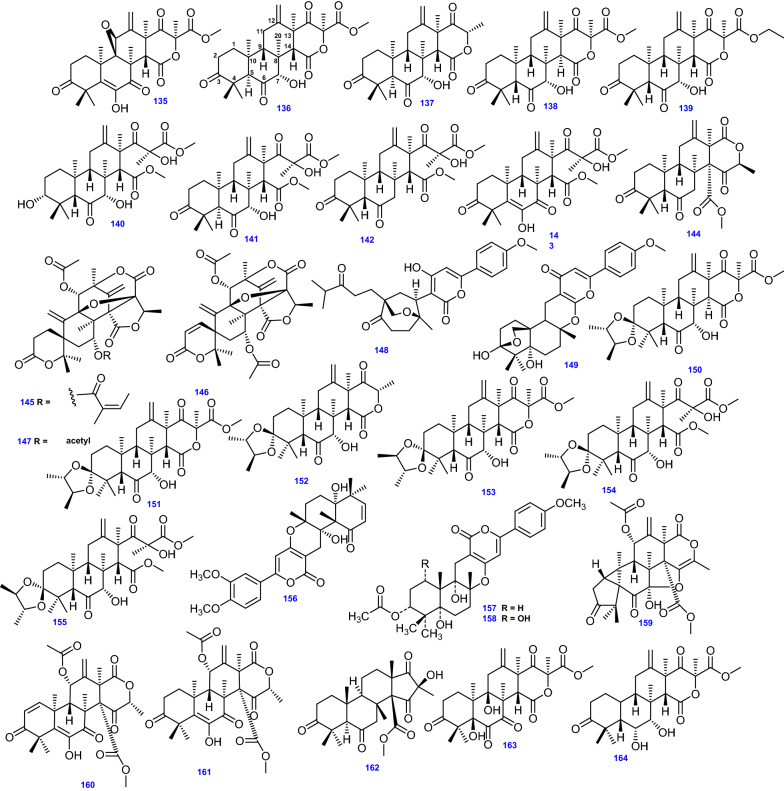

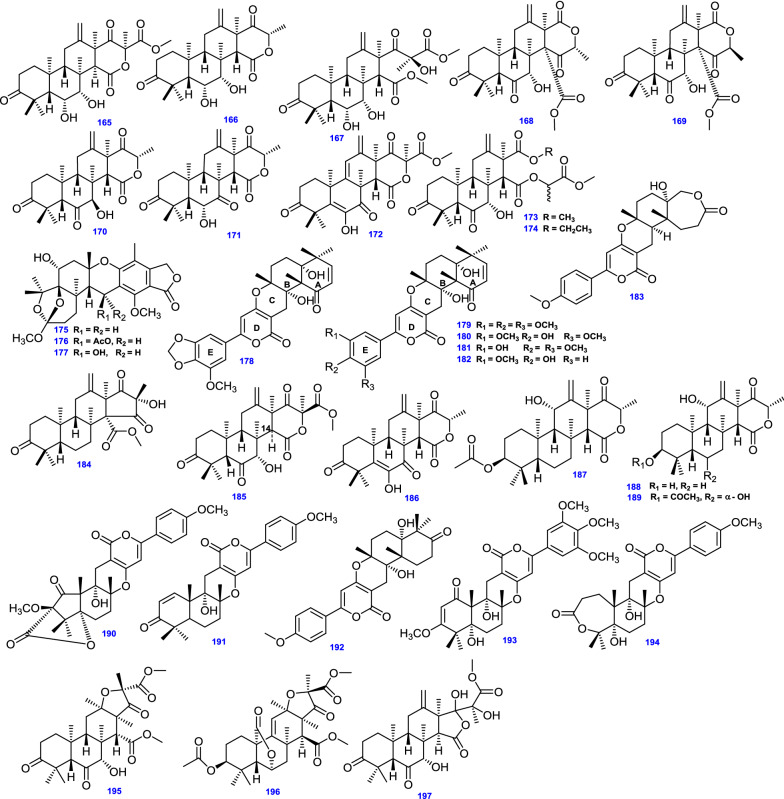
Chemical structures of meroterpenoids obtained from *A. terreus*

All 63 Meroterpenoids (135 -197) isolated from *A. terreus* are compiled in Fig. 12.

### Butenolides and butyrolactones

*Aspergillus* spp. are notable producers of butenolides [85]. Preeminently, the fungus *A. terreus* isolated from both marine and terrestrial sources produce butenolides [86]. Since the first report on the chemical structure of butyrolactone I in 1977, various related phenyl- and benzyl-disubstituted butenolides have been illustrated [85], and fascinatingly, γ-Butyrolactones identified from *Aspergillus* genus, gained a great deal of attention for their bioactivities [69]. This class of butenolides, biogenetically derived from tyrosine and/or phenylalanine, can be classified into three types according to the substitution pattern; 2,3-, 3,4-, and 2,4-disubstituted butenolides [85]. Asperimides (A-D) (198–201) from *A. terreus* SC1550 isolated from *Suriana maritima* L., vary from other butenolides from *Aspergillus* spp. by possessing a maleimide core which extends the chemical scope and biological variability of aromatic butenolides [85].

Terrenolide S (202) has been produced by *A. terreus* obtained from the roots of *Carthamus lanatus* [68]. Other derivatives include 4-(4-hydroxyphenyl)-5-(4-hydroxyphenylmethyl)-2-hydroxyfuran-2-one (203) [40], additionally, 3-[3-hydroxy-4-(3-methyl-but-2-enyl)-phenyl]-5-(4-hydroxybenzyl)-4-methyl-dihydrofuran-2(3H)-one (204), (Z)-3-[3-hydroxy-4-(3-methyl-but-2-enyl) phenyl]-5-(4-hydroxybenzylidene)-4-methyl-dihydrofuran-2(3H)-one (205), and Butyrolactone I (206), were isolated from *A. terreus* obtained from desert soil [121]. Besides, Asperjinone (207), a nor–neolignane compound was derived from *A. terreus* from the soil of the hot springs zone, in Taiwan [43]. Likewise, the butenolide derivatives; 3-hydroxy- 5-[[4-hydroxy-3-(3-methyl-2-buten-1-yl) phenyl] methyl]- 4-(4-hydroxyphenyl)-2(5H)-furanone (208), 5- [(3,4-dihydro-2,2-dimethyl-2H-1-benzopyran-6-yl)-methyl]- 3-hydroxy-4-(4-hydroxyphenyl)-2(5*H*)-furanone (209), Aspernolide A (210) [90], and 4ʹ- dehydroxy aspernolide A (211) were isolated from marine-derived *A. terreus* [122]. Moreover, compounds (208, 209, and 210) were also derived from the salt-tolerant fungus *A. terreus* PT06-2 [64].

A series of aspernolides, Aspernolide B (212) [86], Aspernolide D (213) [68], Aspernolide E (214) [40], Aspernolide F (AF) (215) [69], Aspernolide G (216) [70], Aspernolides N-P (217–219) [123] have been isolated from different *A. terreus* strains. The butenolides containing 5-hydroxyfuran-2(5*H*)-one core; Asperteretals A-C (220–222) were isolated from the endophytic fungus *A. terreus* PR-P-2 derived from *Camellia sinensis* var. *assamica* (Mast.) Kitam [61].

Furthermore, various strains of the endophytic fungus *A. terreus* isolated from different hosts yielded the butenolide derivatives ( ±)-Asperteretal D (223) [87], Asperteretal E (224), and ( ±)-Asperteretal F (225) which contain the 2-benzyl-3-phenyl substituted lactone core [89], Terrein (226) [43] together with the analogues of (223); Flavipesolide B (227), and Flavipesolide C (228), [87]. Marine-derived *A. terreus* produced other derivatives, including 3-hydroxy-4-(4-hydroxyphenyl)-5-methoxycarbonyl-5-(4-hydroxy-3-formylbenzyl)-2,5-dihydro-2-furanone (229) [88], Asperlide A (230) [124], Terrelactone A (231) [64]. On the other hand, Butyrolactone II (232), and Butyrolactone III (233) were obtained from *A. terreus* PTO6-2 isolated from sea sediment [64]. Besides, a series of butyrolactones have been reported from *A. terreus* namely, Butyrolactone IV (234), Butyrolactone V (235) [43], Isobutyrolactone V (236), Isobutyrolactone II (237) [39], 7ʺ-hydroxybutyrolactone III (238) [59], 3ʹ-isoamylene butyrolactone IV (239) [122], Butyrolactone VII (240) [123], and Butyrolactone VIII (241) [90].

It is intriguing to note that, the first butyrolactone possessing α-benzyl and γ-hydroxyl on the unsaturated lactone ring was Butyrolactone VIII (241) which was isolated from *A. terreus* MXH-23. and it could be biosynthesized in the way like Butyrolactone I (206) [30]. Compound (206) was initially isolated from *A. terreus* var. *Africans* IFO 8355 [30], incorporating α-hydroxyl and γ-benzyl substituted lactone ring was biosynthesized by prenylation after the condensation of two *p*-hydroxyphenylpyruvic methyl ester (HPPMe) from phenylalanine [125].

Pulvinones [3-ary1-5-arylidene-4-hydroxyfuran2(5H)-ones] are a group of pigments related to pulvinic acids occurring in lichens and higher fungi [126]. Seto and co-workers discovered dihydroxy-pulvinone derivatives in cultures of *A. terreus* and suggested the name 'aspulvinone' to this family of secondary metabolites to distinguish them from other natural pulvinones [126]. Reported prenylated aspulvinones derivatives from *A. terreus* are Aspulvinone E (242), and Isoaspulvinone E (243) which are photo-interconvertible [91], Aspulvinone H (244) [53], Aspulvinone J-CR (245) [127], Aspulvinone O (246) [82], Aspulvinones R, V–X (247–250) [127], and Pulvic acid (251) [91].

On the chemical assessments of marine endophytic *A. terreus* obtained from the inner part of the soft coral *Sarcophyton subviride*, other butenolides have been isolated; namely, 8ʺ *R*,9ʺ -diol versicolactone B (252), 8ʺ *S*,9ʺ -diol versicolactone B (253), Versicolactone B (254) [122], and Versicolactone G (255) [45]. The antifungal butanolide, Sinulolide I (256) was isolated as a fatty acid derivative from the endophytic fungus *A. terreus* SCSIO 41202 obtained from deep-sea sediment [66]. Besides, four butenolide metabolites, Terrusnolides A − D (257–260), were isolated from *A. terreus* obtained from the root of *Tripterygium wilfordii* [128]. It is worth noting that Terrusnolide A (257) was biosynthesized through a triple decarboxylation, whereas (258–260) comprised a 4-benzyl-3-phenyl-5H-furan-2-one moiety having an isopentene group fused to the benzene ring [128].

**Fig. 13:**
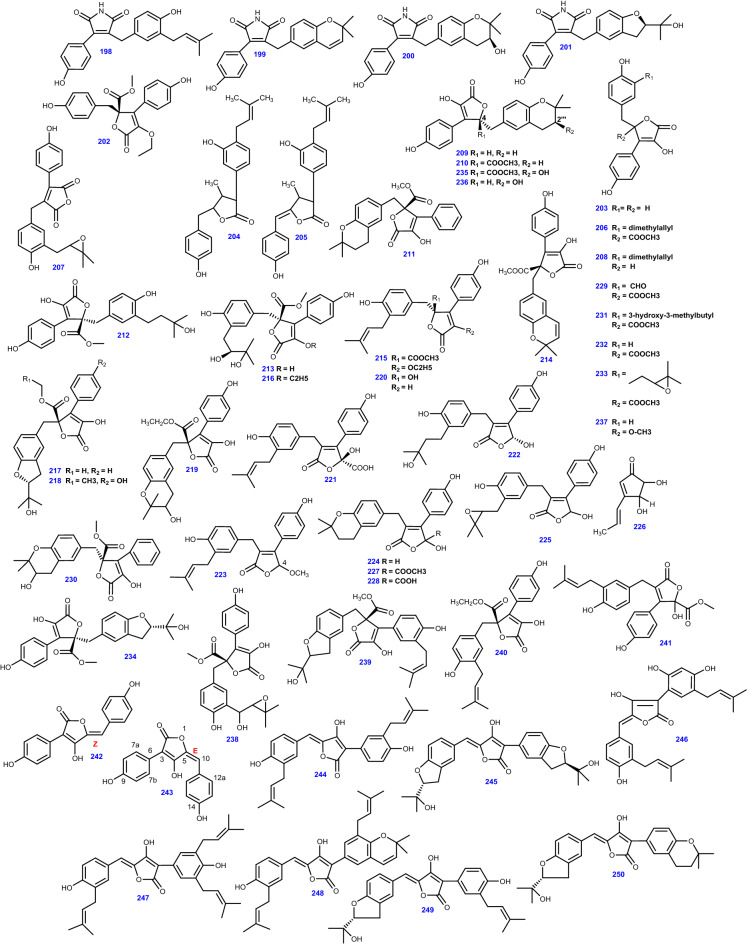

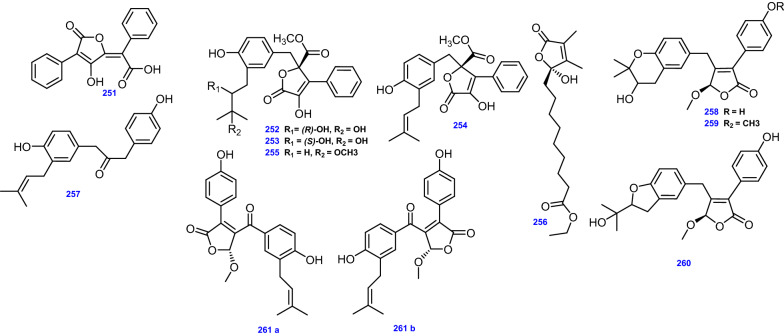
Chemical structures of Butenolides and Butyrolactones isolated from *A. terreus*

Butenolide derivatives, ( ±)-Asperteretone F (261 a/261 b) were obtained from the endophytic fungus *A. terreus* isolated from *Hypericum perforatum* collected from the Muyu Town in the Shennongjia region, Hubei Province, People’s Republic of China [92]. All 64 Butenolides and Butyrolactones (198–261) from *A.terreus* are illustrated in Fig. 13.

### Polyketides

A thiophene naphthoquinone derivative, (8-hydroxy-2-[1-hydroxyethyl]-5,7-dimethoxynaphtho[2,3-b] thiophene-4,9-dione) (262) has been isolated from the mangrove *Bruguiera gymnoihiza* ( Linn.) savigny-associated *A. terreus*, together with Anhydrojavanicin (263), 8-*O*-methyljavanicin (264), Botryosphaerone D (265), and 6-ethyl-5-hydroxy-3,7-dimethoxynaphthoquinone (266) [65].

The thermophilic endophytic fungus *A. terreus* (C9408-3) yielded the chlorinated diphenyl ether, Geodin hydrate (267), the dichloro-benzophenone derivatives, Dihydrogeodin (268), 2-(3,5-dichloro-2,6-dihydroxy-4-methylbenzoyl)-5-hydroxy-3-methoxybenzoic acid (269), *ω*-Hydroxyemodin-5-methyl ether (270), *ω*-Acetylcarviolin (271), Questin (272), Methyl 3,5-dichloroasterric acid (273), as well as Asterric acid (274) [43].

A number of polyketides such as Rhizoctonic acid (275), and Monometylsulochrin (276), were produced from the endophytic fungus *A. terreus* isolated from stem of rice [47]. Similarly, the anthraquinone derivatives; 1,8-dihydroxy-3-methoxy-6-methylanthracene-9,10-dione (277), and 1-methyl emodin (278), plus the naphthalenoid derivatives; Methyl 6-acetyl-4-methoxy-5,8-dihydroxynaphthalene-2-carboxylate (279), and Methyl 6-acetyl-4-methoxy-5,7,8-trihydroxynaphthalene-2-carboxylate (280) were isolated from *A. terreus* SCSIO 41008 associated to marine *Callyspongia* sp. [41]. The spiroketal derivative, Aspergiketal (281), and the anthraquinone Physcion (282) were isolated from *A. terreus* obtained from the fresh stems of *Opuntia ficus indica* Mill [98]. Furthermore, by utilizing bioassay-guided fractionation, *A. terreus* Thom derived from the rhizosphere of a staghorn cholla (*Opuntia versicolor* Engelm.) provided the quinone derivative, Betulinan A (283) [48]. In addition, Emodin (284), and the naphthalenoid derivative, Methyl 6-acetyl-4-methoxy-7,8-dihydroxynaphthalene-2-carboxylate (285) were separated from *A. terreus* QT122 isolated from mature dragonfly [99].

Moreover, Cowabenzophenone A (286) was derived from *A. terreus* associated with the mangrove *Bruguiera gymnorrhyza* [100]. In addition, the xanthone derivative; Penicillixanthone (287), the mono-chloro-benzophenone derivative; Monochlorosulochrin (288), and the dichloro-benzophenone derivative; NP-002561 (289) were derived from the marine-derived fungus *A. terreus* obtained from the coral *Echinogorgia aurantiaca* [38].

**Fig. 14:**
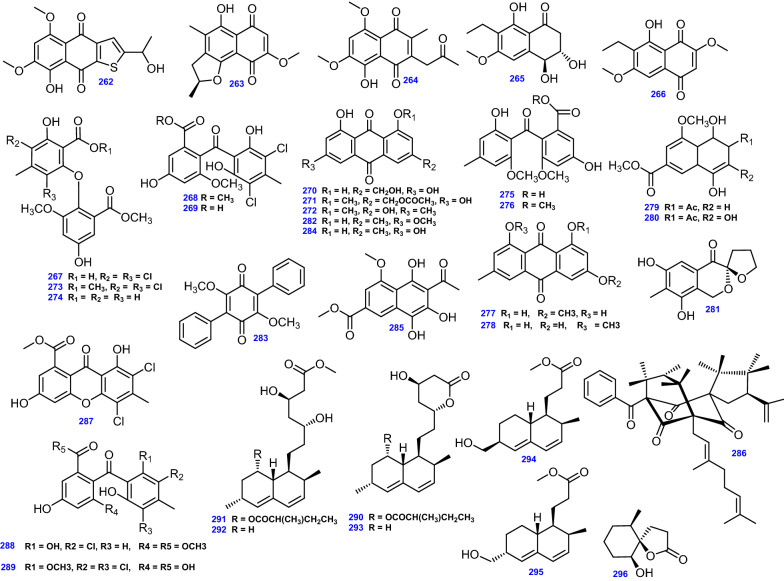
Chemical structures of polyketides compounds isolated from *A. terreus*

Statins are polyketide molecules that are produced by some fungi in the course of their secondary metabolism [129]. The polyketide fatty acid ester derivatives including, the antilipemic agent, Lovastatin (Monacolin K) (290) [45], Methyl ester of lactone ring-opened monacolin K (291) [130], Monacolin L acid methyl ester (292) and Monacolin L (293) were derived from the marine-derived endophytic *A. terreus* [45]. Whereas two lovastatin analogues, Terrstatins A and B (294–295, respectively) were afforded by *A. terreus* which derived from the *Hypericum perforatum* flower [92]. The spirocyclic lactone, Terreinlactone C (296) was identified as the first naturally occurring compound possessing a 1-oxaspiro[4.5]decan-2-one ring structure from *A. terreus* [105]. Compound (296) also represented a novel type of polyketide in addition to the well-known type terreins [105]. All 35 polyketide compounds (262- 296) from *A. terreus* are depicted in Fig. 14.

### Miscellaneous

Researchers from Hoechst Marion Roussel Deutschland GmbH (now SanofiAventis) isolated the antihyperglycemic agents, Kodaistatins A–D (297–300) from soil derived *A. terreus*, which incorporate a pulvinone unit, a dienone side chain with one stereocenter, and a dihydroxycyclopentenone core with two stereocenters [93, 94]. Additionally, chemical evaluation of *A. terreus* yielded a quinone compound, Terreic acid (301) [35], phenol derivative, 4,5-dimethylresorcinol (302) [53], and *P*-hydroxy-benzoic acid (303) [50]. Conjointly, the prenylated phenol derivatives; Terreprenphenol A (304), Terreprenphenol B (305), Terreprenphenol C (306), 4-hydroxy-3-prenybenzoic acid (307), and 4-hydroxy-3-(3-methyl-but-2-enyl)-benzaldehyde (308) were isolated from the endophytic fungus *A. terreus* obtained from the marine red alga *Laurencia okamurai* [78]. Interestingly, compound (304) was a prenylated phenol derivative that resembled 4- hydroxy-3-(3-methyl-2-butenyl) acetophenone (HMBA), the key secondary metabolite of *Senecio nutans* (Asteraceae) [78]. *P*-hydroxybenzaldehyde (309), *P*-hydroxyphenylacetic acid methyl ester (310), *O*-hydroxyphenylacetic acid methyl ester (311), and Kojic acid (312) have been isolated from marine soft coral-derived *A. terreus* SCSIO41404 [130]. Moreover, four dihydrobenzofuran derivatives, Anodendroic acid (313) [78], and Asperterreusines A-C (314–316) were reported in *A. terreus* [72].

On the other hand, a tetrahydroxybenzaldehyde derivative, FR198248 (4-methyl-l,3-dihydro-2-benzofuran1,5,6,7-tetraol) (317), was obtained from the culture broth of *A. terreus* 13830 [95]. Besides, chromatographic fractionation of the crude methanolic extract of *A. terreus* ZDF21 from a soil sample afforded citrinin dimers, Dicitrinin A (318), and Dicitrinin E (319), along with their monomer, Citrinin (320) [58].

The isocoumarin derivatives; *R* ( −)-6-hydroxymellein (321), and *Trans*-4,6-dihydroxymellein (322) were derived from the salt-tolerant fungus *A. terreus* obtained from sea sediment [64]. Similarly, from *A. terreus* derived from marine sediments, a series of bioactive isocoumarin derivatives were obtained: 6-(4’-hydroxy-2’-methyl phenoxy)-(–)-(3R)-mellein (323), (–)-(3*R*)-6-methoxymellein (324), (–)-(3*R*)-6,7-dimethoxymellein (Kigelin) (325), and (3*R*, 4*R*)-6,7-dimethoxy-4-hydroxymellein (326) [97]. Additionally, (*S*)-6, 8-dimethoxy-3-methylisochroman-1-one (327) was derived from *A. terreus* SCSIO 41008 isolated from the marine *Callyspongia* sp. [41].

Fatty acids and fatty acid methyl ester derivatives have been reported from *A. terreus* such as Linoleic acid (328), which was isolated from *A. terreus* from a sub-surface soil sample in Egypt [50], whereas Oleic acid (329) was obtained from the endophytic fungus *A. terreus,* var. *boedijnii* (Blochwitz) originated from red marine alga *Laurencia ceylanica*, J. Agardh [88], and Methyl linoleate (330) from the extremophilic *A. terreus* LGO13 [82]. On the other hand, Dodecanoic acid (331), and Decanoic acid (332) were isolated by bioassay-guided fractionation of EtOAc extract of the deep-sea sediment-derived *A. terreus* [66], together with Decanoic acid (2,2-dimethyl-1,3-dioxolan-4-yl) methyl ester (333) [53]. Besides, a linear aliphatic alcohol, (3*E*,7*E*)-4,8-dimethyl-undecane-3,7-diene-1,11-diol (334) was isolated from marine-derived *A. terreus* [45], and *R* (–)-glycerol monolinoleate (335) was isolated from soil fungus *A. terreus* [53]. D-mannitol (336) was obtained on the chemical examination of *A. terreus* derived from a garbage component at an organic fertilizer factory, (Thailand) [67].

Moreover, two furandione derivatives, Asperterone B (337),and Asperterone C (338) were reported from the liquid culture of *A. terreus* MHL-P22 isolated from *Malus halliana* [57].

Another furandione derivative; Asperterone (339) was isolated from *A. terreus* obtained from *Mammea siamensis* [86]. Among curvularin group, three compounds; Dehydrocurvularin (340), 11-methoxycurvularin (341), and 11- hydroxycurvularin (342) were produced by *A. terreus* occurring in the rhizosphere of a *Brickellia* sp. [49]. The furopyran metabolite, Patulin, (343) has been reported from *A. terreus* [35]. He et al. discovered the furan-containing compound, Terrefuranone (344) through a chemical analysis of *A. terreus* isolated from the rhizosphere of the canyon ragweed [*Ambrosia ambrosioides* (Cav.) Payne; Asteraceae] [49]. In addition to the benzopyran derivative, 2,2-dimethyl-3-hydroxychroman-6-aldehyde (345) was isolated from *A. terreus* [105].

**Fig. 15:**
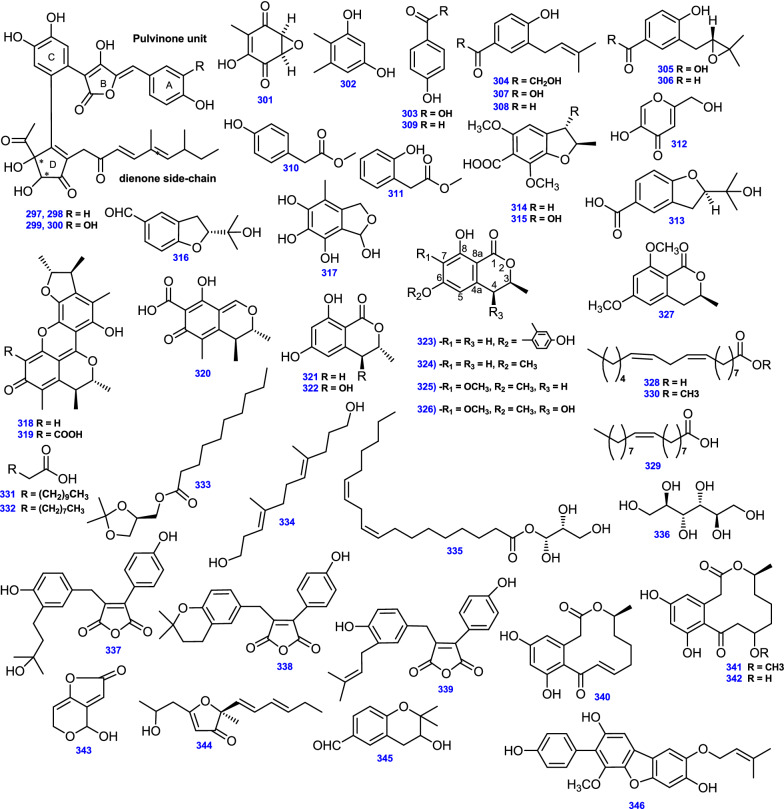
Chemical structures of miscellaneous compounds isolated from *A. terreus*

A known terphenyl-type metabolite, Arenarin A (346) was first discovered from sclerotia of *Aspergillus arenarius* (NRRL 5012) [131], and has been reported from *A. terreus* isolated from *Ipomoea carnea* (Convolvulaceae) [106]. All 50 Miscellaneous compounds (297- 346) from *A. terreus* are depicted in Fig. 15.

**Fig. 16:**
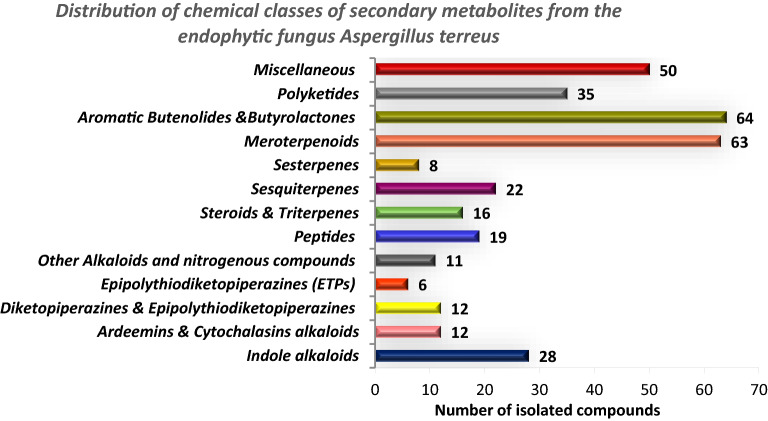
A bar chart for major chemical classes of metabolites produced by *A. terreus*, showing butenolide—butyrolactone class and meroterpenoid class as principal classes

**Fig. 17:**
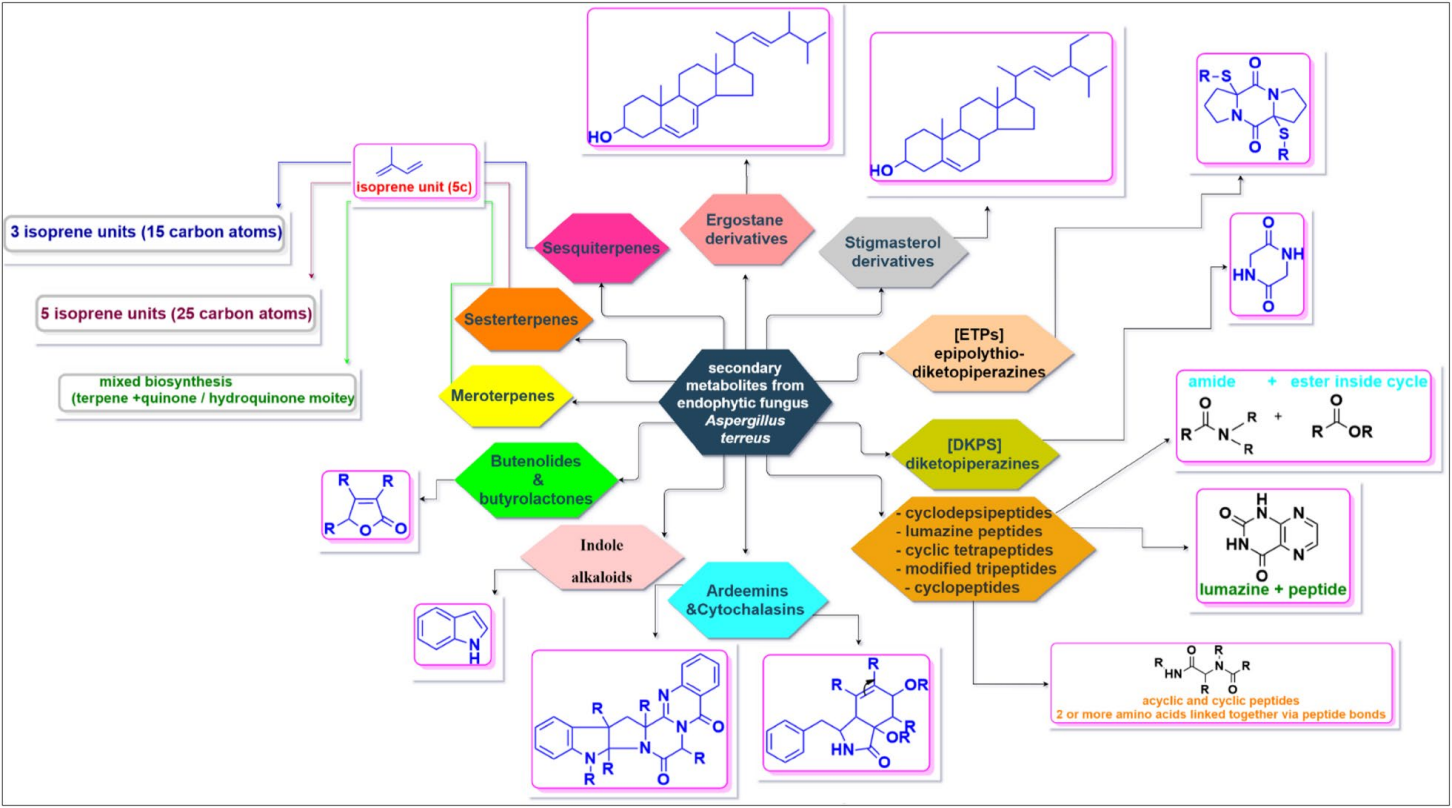
Synopsis for common chemical nucleus and the main building blocks of the secondary metabolites produced by the endophytic fungus *A. terreus*

In an attempt to provide information about the main chemical classes produced by *A. terreus*, among the 346 secondary metabolites isolated, 63 were meroterpenoids and 64 were butenolides—butyrolactones, suggesting that the butenolide—butyrolactone class and the meroterpenoid class are two major chemical classes, as depicted Fig. 16, with a summary of the most common chemical nuclei and main building blocks of the secondary metabolites produced by the endophytic *A. terreus* as presented in Fig. 17.

## Biological investigation

### Antimicrobial activity

#### Anti-mycobacterium tuberculosis (TB) activity

Butyrolactone I (206) was found to exhibit powerful Mycobacterial protein tyrosine phosphatase B (MptpB) inhibitory activity with a half maximal inhibitory concentration (IC_50_) of 5.11 µM, compared to oleanolic acid, (22.1 µM) [41]. Besides, it revealed weak or no cytotoxic activities towards human glioma U87 cells at a concentration of 10 µM.

Moreover, Bisdethiodi(methylthio)-acetylaranotin (56) was found to be the antimycobacterial principle from *A. terreus* BCC 4651 showing minimum inhibitory concentration (MIC) value of 1.56 μg/mL against *M. tuberculosis* H37Ra, compared to Isoniazid (MIC value of 0.05 μg/mL) [63]. However, Bisdethiobis(methylsulfanyl)apoaranotin (58) displayed poor activity (MIC = 25 μg/ mL) [62].

#### Anti-viral activity

Some of the butenolide derivatives were reported to inhibit influenza H1N1 in vitro, with a half maximal effective concentration (EC_**50**_) of 6.7 µM [132]. Besides, some butenolides were potent antiviral agents against hepatitis B and C viruses [132]. Butyrolactone I (206) could be a promising drug candidate for the influenza virus demonstrating anti-H1N1 activity with IC_50_ and CC_50_ values of 143.1 and 976.4 µM, respectively (positive control: ribavirin, IC_50_ 100.8 µM). Furthermore, compound (206) displayed kinase inhibition with high selectivity towards cyclin-dependent kinase 1 (CDK1) and cyclin-dependent kinase 2 (CDK2) [64].

The meroterpenoid Aperterpene N (162) demonstrated inhibitory activity towards influenza neuraminidase (NA) with an IC_50_ value of 18 mM [77]. This enzyme is associated with provoking the release of descendant viruses from the surface of infected cells and is believed to promote viral movement across the respiratory tract [95]. An anti-influenza FR198248 (317), isolated from the endophytic fungus *A. terreus*, was involved in the screening of novel viral neuraminidase inhibitors [95]. Intriguingly, monomethylated products of (317), (1-Methoxy-4-methyl- 1,3-dihydro-2-benzofuran-5,6,7-triol and 6-Methoxy-4-methyl- 1,3 -dihydro-2-benzofuran- 1,5,7-triol) were equipotent as (317), which denotes all hydroxyl groups are not essential for the activity of (317) [95]. The benzyl alcohol derivative; 2,3,4-Trihydroxy-6-(hydroxymethyl)-5-methylbenzylalcohol, the reduced form of (317), exhibited marginally poorer anti-influenza activities than (317), yet the antiviral activity was still maintained, indicating that some hydroxyl-benzyl alcohol derivatives are potential antiviral agent [95]. Notwithstanding, either methylation or acetylation of (317) dramatically decreased the activity, suggesting that phenolic hydroxyl groups had a crucial role in activity [95]. FR198248 (317), not only displayed powerful anti-influenza A and B activity in vitro (comparable to ribavirin) but also showed potent activity in vivo [96].

While utilizing plaque reduction assay to assess the antiviral activity against HSV-1, compounds 11a-dehydroxyisoterreulactone A (183), Arisugacin A (156), Isobutyrolactone II (237), and Aspernolide A (210), had an antiviral activity with IC_50_ of 16.4, 6.34, 21.8, and 28.9 μg/mL, respectively, under their non-cytotoxic concentrations against Vero cell line [39]. Besides, the butenolides Pulvic acid (251), Aspulvinone E (242), and Isoaspulvinone E (243), exhibited marked anti-influenza A H1N1 virus activities, with IC_50_ values of 29.1, 56.9, and 32.3 μg/mL, respectively. Additionally, compound (243) with *E* ∆^5(10)^ displayed remarkable inhibitory activity against H1N1 viral NA [91]. Docking of two isomers (242 & 243) into the active sites of NA revealed that the *E* double bond ∆^5(10)^ was crucial for the activity. These results hold promise to afford new antiviral chemotypes to restrain influenza infection [91].

In addition, Butyrolactone III (233) and 5- [(3,4-dihydro-2,2-dimethyl-2*H*-1-benzopyran-6-yl)-methyl]- 3-hydroxy-4-(4-hydroxyphenyl)-2(5*H*)-furanone (209) displayed moderate inhibition rate 53.9 and 57.8%, respectively, against influenza H1N1 virus at concentration 50 μg/L, versus an inhibition rate of 78.0% of ribavirin [90].

The cyclic tetrapeptide, Asperterrestide A (71) displayed inhibitory activity against H1N1 influenza virus strain A/WSN/33 (an M2-resistant strain) and the H3N2 strain A/Hong Kong/8/68 (an M2-sensitive strain) with IC_50_ values of 15 and 8.1 μM, respectively versus 20.2 and 0.41 μM, respectively, for the standard (RIBA) [40].

#### Anti-bacterial activity

The terpenoidal secondary metabolite, Amhezole (104), demonstrated significant inhibition against microbial mouth infections; caused by *Lactobacillus acidophilus*, *Streptococcus gordonii*, and *Streptococcus mutans* [71]. The combination of compound (104) with Coe-Comfort tissue conditioner suppressed the growth of *L. acidophilus* at a concentration of 7.81 μg/mL, *S. gordonii* at a concentration of 62.50 μg/mL, and *S. mutans* at a concentration of 125 μg/mL [71]. Interestingly, the oral administration of the compound (104) in the toxicity study did not significantly affect the activity of alanine aminotransferase, aspartate aminotransferase, and the levels of blood urea and serum creatinine [71].

While Aspernolide F (AF) (215) exhibited poor activity against Methicillin-resistant Staphylococcus aureus (MRSA) (IC_50_ = 6.39 mg/mL), the stigmasterol derivative (22*E*,24*R*)-stigmasta-5,7,22-trien-3-β-ol (89) demonstrated a remarkable activity with IC_50_ value as low as 0.96 μg/mL compared to ciprofloxacin (IC_50_ value = 0.07 μg/mL) [70]. Terretonin G (134) demonstrated inhibitory activity against *Micrococcus luteus* and *Staphylococcus aureus*, with MIC values of 32 and 8 μg/mL, respectively (chloramphenicol MIC = 1 μg/mL), recommending that ring D hydrolysis in terretonins could improve their antimicrobial activities [77]. The butenolides, Asperteretal E (224), and Aspernolide A (210) displayed moderate antimicrobial activities against *S. aureus, Bacillus thuringiensis, Bacillus subtilis*, and *Escherichiacoli* with inhibition zone diameters of 8.94, 9.77,7.98 and 7.53 mm and 8.16, 9.13, 7.49, and 7.64 mm, respectively [89].

Furthermore, remarkable antibacterial activity against six microbial pathogens; ESBL-producing *E. coli*, *Acinetobacter baumannii, Pseudomonas aeruginosa, Klebsiella pneumoniae,* MRSA, and *Enterococcus faecalis* was demonstrated by Asperteramide A (68) with MIC values of 8, 8, 16, 64, 64, 8 μg/mL, respectively [46]. Aspulvinone H (244) and *R* (–)-glycerol monolinoleate (335) exhibited antibacterial activities against *S. aureus* and *B. subtilis* with MIC of (8, 64) and (32, 32) μg/mL, respectively, comparable to the positive controls, nystatin, and kanamycin [53]. Butyrolactone I (206) efficaciously suppressed the growth of *E. coli* (ATCC 25,922) and killed it at 117.6 μM, whereas tetracycline, the positive control, carried out these activities at 7 µM [102].

Antibacterial activities of Terreprenphenols A–C (304–306), 4-hydroxy-3-prenybenzoic acid (307), 4-hydroxy-3-(3-methyl-but-2-enyl)-benzaldehyde (308), Anodendroic acid (313) and Asperterreusine C (316) were assessed against the human pathogens; *E. coli* and *S. aureus* and the aquatic bacteria; *Aeromonas hydrophila, Edwardsiella tarda*, *Micrococcus luteus, Pseudomonas aeruginosa, Vibrio harveyi*, *Vibrio parahaemolyticus*, and *Vibrio vulnificus* where compounds (304), (307), and (308), displayed broad-spectrum inhibitory activity against the pathogenic bacteria [78]. Compound (304) demonstrated remarkable antibacterial action specifically towards the aquatic bacteria *A. hydrophila, P. aeruginosa., and V. harveyi* with MIC values = 2, 2, and 4 µg/mL, respectively, and moderate to poor towards *E. tarda, E. coli, M. luteus, S. aureus, V. parahaemolyticus and V. vulnificus* with MIC values = 32, 32, 16, 8, 8, and 32 µg/mL, respectively [78]. However, (307) showed moderate to weak activity towards *A. hydrophila, E. tarda, E. coli, S. aureus, V. harveyi, V. parahaemolyticus* with MIC value = 8, 16, 16, 64, 32, and 8 µg/mL, respectively [78]. Furthermore, (308) displayed potent activity towards *A. hydrophila* with MIC values = 4 µg/mL, whereas moderate to poor towards *E. tarda, E. coli, M. luteus, P. aeruginosa, S. aureus, V. harveyi, V. parahaemolyticus and V. vulnificus* with MIC values = 16, 32, 8, 16, 16, 8, 8, and 64 µg/mL, respectively [78]. Accordingly, the prenyl group was found to be substantial for antibacterial activity (304, 307, 308 vs. 305, 306, 313, and 316) [78].

In disc diffusion assay, promising antibacterial activity against the Gram-positive foodborne bacteria *Bacillus cereus, B. spizizenii, B. subtilis* and *S. aureus* has been demonstrated by the EtOAc extract of the endophytic fungus *A. terreus* MP15, compound Di-n-octyl phthalate can make a significant contribution to this antibacterial activity and can possibly be a food preservative and colorant [103].

Cowabenzophenone A (286) displayed potent selective antibacterial activity against *B. subtilis* (UBC 344), and *S. aureus* (ATCC 43,300) with MIC values of 1 and 2 μg/mL respectively, compared to Polymixin B (MIC = 4 μg/mL), and Rifamycin (MIC = 1 μg/mL), respectively. In addition, it demonstrated promising activity against MRSA (ATCC 33,591), with MIC of 4 μg/mL, compared to 1 μg/mL for Rifamycin. It also exhibited activity against the Gram-negative strains *E. coli* (UBC 8161) and *P. aeruginosa* (ATCC 27,853), with MIC of 4 and 2 μg/mL, respectively, versus 1 μg/mL for Polymixin B [100]. Moreover, Terreic acid (301) and Butyrolactone I (206) showed activity towards the phytopathogenic bacteria *Erwinia carotovora* with IC_50_ values of 5.1 and 12.5 μg/mL, respectively, whereas the standard drug, streptomycin displayed IC_50_ of 1.9 μg/mL [101].

#### Antifungal activity

Butyrolactones have a broad scope of biological activities [70]. The butyrolactone, Aspernolide F (AF) (215), exhibited remarkable activity against *Cryptococcus neoformans* (IC_**50**_ = 5.19 μg/mL) [70]. Similarly, the butenolide, Sinulolide I (256), along with the fatty acid derivative (9*Z*,12*Z*)-*N*-(2-hydroxyethyl) octadeca-9,12-dienamide (69), Dodecanoic acid (331), and Decanoic acid (332) displayed remarkable antifungal activity against the phytopathogenic fungus *Penicillium italicum* which causes postharvest disease in citrus fruits, with MIC values of 0.125, 0.062, 0.031 and 0.062 mg/mL, respectively, showing great applicability as natural fungicides [66]. However, the Stigmasterol derivative (22E,24R)-stigmasta-5,7,22-trien-3-β-ol (89), revealed activity against *C. neoformans* with IC_50_ value of 4.38 mg/mL compared to Amphotericin B (IC_**50**_ = 0.34 μg/mL), [70]. Amhezole (104), displayed considerable action against fungal mouth infection, and its combination with Coe-comfort tissue conditioner suppressed the growth of *Candida albicans* at a low value of the MIC equal to 7.81 μg/mL [71].

On the other hand, compounds 1-methyl emodin (278) and Terrein (226) presented powerful antifungal activity against *Alternaria solani* with IC_**50**_ < 0.1 μg/mL, and the compound (278) also possessed a potent inhibitory effect towards the growth of *Fusarium oxysporum f. sp. cucumerinum* (IC_50_ < 0.1 μg/mL), comparable to the positive control cycloheximide while compound (251) showed moderate activity (IC_50_ = 5.7 μg/mL) [99]. Besides, both compounds exhibited moderate antifungal activity against *F. graminearum* with IC_50_ values of 19.1 and 0.6 μg/mL, respectively [99].

Butyrolactone I derivatives; 3-[3-hydroxy-4-(3-methyl-but-2-enyl)-phenyl]-5-(-4-hydroxybenzyl)-4-methyldihydrofuran-2(3H)-one (204) and (Z)-3-[3-hydroxy-4-(3-methyl-but-2-enyl)-phenyl]-5-(-4-hydroxybenzylidene)-4-methyl-dihydrofuran-2(3H)-one (205), showed antifungal activity against *Aspergillus fumigatus* with MIC of 34.13 and 17.06 μg/mL, respectively [121].

In addition, 6-(4’-hydroxy-2’-methyl phenoxy)-(–)-(3R)-mellein (323) and (3R, 4R)-6,7-dimethoxy-4-hydroxymellein (326) demonstrated remarkable activity against the human pathogenic dermatophytes; *Trichophyton longifusus,* and *Microsporum canis* at a concentration of 200 µg/mL, with inhibition percentages of (55–70%) and (70% and 50%) respectively, while miconazole achieved complete inhibition at 70 and 98.4 μg/mL, respectively [97]. The crude extract of *A. terreus* derived from *Morus indica* leaf possessed promising antifungal activity against *Macrophomina phaseolina* and Mulberry charcoal rot pathogen [104].

Physcion (282), a natural antifungal that is used to treat plant diseases, including downy mildew, powdery mildew, grey mould, and other fungal infections on plants, has tremendously promising biopesticidal applications and has been developed and marketed in China as a fungicide [133–135].

### Antifouling activity

Compounds ($-$)-Alantrypinone (74), Methyl 3,4,5-trimethoxy-2-(2-(nicotinamido)benzamido) benzoate (61), Penicillixanthone (287), had remarkable antifouling activity against larvae of the barnacle *Balanus amphitrite*, with EC_50_ of 17.1, 11.6, and 17.1 μg/mL, respectively, and LC_50_/EC_50_ values > 100 [38]. However, compounds Dihydrogeodin (268), Monochlorosulochrin (288), NP-002561 (289), and Methyl dichloroasterrate (328) showed poor or no activity, suggesting that the benzopyranone moiety may play a vital role in the antifouling activity [38].

Additionally, compounds Territrem D (181), Butyrolactone V (235), Aspernolide A (210), and Aspernolide B (212) are promising natural nontoxic antifouling agents, with powerful antifouling activity at nontoxic concentrations (LC_50_/EC_50_ values > 100 μg/mL) with EC_50_ values of 12.9, 22.1, 7.4, and 16.1 μg/mL, respectively, against barnacle *Balanus amphitrite* larvae [39]. Natural antifoulants should have an EC_50_ level of 25 μg/mL, and usually, an antifouling compound with LC_50_/EC_50_ > 15 is considered safe [39]. Compounds (181), (235), (210), and (212) are potentially safe and promising antifouling agents [39].

### Antiparasitic activity

#### Anti-leishmanial activity

The stigmasterol derivatives (22*E*,24*R*)-stigmasta-5,7,22-trien-3-β-ol (89), and Stigmast-4-ene-3-one (90) together with the butenolide derivative Terrenolide S (202) exerted exceptional anti-leishmanial activity against *Leishmania donovani* with IC_50_ of 11.24, 15.32 and 27.27 μM, respectively, and IC_90_ of 14.68, 40.56 and 167.03 μM, respectively, in comparison with the positive control pentamidine (IC_50_ = 6.18 μM and IC_90_ 28.15 μM) [68]. Compounds Terrein (226), Butyrolactone V (235) and Butyrolactone I (206) displayed moderate leishmanicidal activity against *L. amazonensis* with IC_50_ of 78.6, 23.7, and 26.0 μM, respectively, compared to the positive control amphotericin B (IC_50_ of 0.2 μM) [102].

#### Anti-schistosomal activity

It has been found that the EtOAc extract of the endophytic fungus *A. terreus* -F7 from *Hyptis suaveolens* (L.) Poit and the isolated compounds Terrein (226), Butyrolactone V(235), and Butyrolactone I (206) demonstrated schistosomicidal activity against *Schistosoma mansoni* adult worms. Both Praziquantel (positive control) and the EtOAc extract killed 100% of the worms at concentrations of 12.5 µM and 100 µg/mL, respectively, after 24 h [102]. Terrein (226) and Butyrolactone I (206) achieved the same result but at concentrations 1297.3 and 471.2 µM, respectively, after 48 h [102]. The extract produced the best outcome presumably because of the synergistic interaction of the metabolites [102].

#### Anti-plasmodial activity

Territrem B (179) demonstrated antiplasmodial activity against *Plasmodium falciparum* with an IC_50_ value of 2.83 μg/mL [67]. Likewise, Butyrolactone V (235) displayed antiplasmodial activity against *P. falciparum* K1 with an IC_50_ of 7.9 μg/mL, whereas the standard antimalarial dihydroartemisinin had an IC_50_ value of 0.0011 μg/mL [63].

#### Anti-filarial activity

Cowabenzophenone A (286) displayed exceptional anti-filarial activity with MIC, IC_50,_ and median lethal concentration (LC_50_) values as low as 0.358 mg/mL, 0.708 mg/mL, and 3.89 mg/mL, respectively, compared to Ivermectin (IVM) (MIC, IC_50_ and LC_50_ values of 3.12, 6.25, and 16.57 mg/mL, respectively) against microfilariae and adults [100].

### Mosquitocidal activity

The assessment of the histopathology, smoke toxicity effect, neurobehavioral toxicity, knock-down efficacy, as well as the ovicidal and adulticidal effects of *A. terreus* extract on three mosquito species: *Culex quinquefasciatus*, *Anopheles stephensi*, and *Aedes aegypti* (Diptera: Culicidae), proposed that the *A. terreus* isolates could be environmentally friendly, cost-effective, and target specific mosquitocidal tool in the future [136]. The biochemical investigation revealed a decline in the level of acetylcholinesterase, α-carboxylesterase, and β-carboxylesterase in extract-treated larvae of all tested mosquito species [136]. Histopathological examination revealed the disorganization of the abdominal region, loss of antenna, lateral hair, caudal hair, and upper and lower head hairs in extract-treated *A. stephensi, C. quinquefasciatus*, and *A. aegypti* [136]. In addition, dose-dependent inhibition of mosquito hatchability percentage was observed with *A. terreus* extract [136]. At 500 μg/mL concentration, the hatchability of mosquito eggs was zero [136]. The EtOAc extract had the best adulticidal activity against *A. stephensi, and C. quinquefasciatus* followed by *A. aegypti* with no mortality in the control group [136]. Moreover, the results of the smoke toxicity assay of the mycelia extract reported a substantial mortality rate towards *A. aegypti* (91%), *C. quinquefasciatus* (89%), and *A. stephensi* (84%) [136].

### Anti-inflammatory and immunomodulatory activity

Yaminterritrems B (149), a meroterpenoid isolated from *A. terreus* displayed a dose-dependent inhibitory effect on cyclooxygenase-2 (COX-2) expression in lipopolysaccharide (LPS)-stimulated RAW264.7 macrophages at protein and RNA levels with an EC_50_ value of 18.3 μM, [42].

Additionally, Brasilanones A (108) and E (112) reduced nitric oxide (NO) production with inhibition rates of 47.7–7.3% at the concentration of 40 μM [72]. Conjointly, Aspermeroterpenes A, B, and C (159,160,161) demonstrated notable inhibitory activities towards LPS-stimulated NO production in RAW 264.7 cells with IC_50_ values = 17.8, 14.1, and 13.4 μM superior to indomethacin (IC_50_ = 24.0 μM) [80].

The butenolide Aspernolide A (210), Asperteretal A (220), Asperteretal C (222), Butyrolactone II (232), and Butyrolactone III (233) exhibited powerful inhibitory effects on NO production in RAW 264.7 LPS -stimulated macrophages with IC_50_ values of 45.37, 26.64, 16.80, 44.37, and 20.60 μM, respectively, superior to hydrocortisone (IC_**50**_ = 48.66 μM) [61]. However, Terretonin A (129), Terretonin D (132), Terretonin (133), Terretonin D1 (185), (50 μg/mL) demonstrated weak inhibitory effects on NO production with inhibition percentage of 22.5%, 23.5%, 34.0%, and 30.2%, respectively, [81]. Also, compounds Luteoride E (2), Methyl 3,4,5-trimethoxy-2-(2-(nicotinamido) benzamido) benzoate (61), 14*α*-hydroxyergosta-4,7,22-triene-3,6-dione (95), Territrem A (178), Versicolactone G (255), (3E,7E)-4,8-dimethyl-undecane-3,7-diene-1,11-diol (334), and Lovastatin (290) proved important anti-inflammatory activity against NO production with IC_50_ values of 24.64, 5.48, 26.83, 29.34, 15.72, 18.62, and 17.45 μM, respectively [45].

Aspernolide F (AF) (215) is a cardioprotective butyrolactone isolated from the endophytic fungus *A. terreus,* AF efficiently protected against doxorubicin (DOX)- induced cardiac damage as AF hindered DOX-induced electrocardiogram (ECG) abnormalities and weakened serum markers of cardiotoxicity (creatine kinase-MB, lactate dehydrogenase, troponin I, and troponin T). Additionally, AF significantly improved DOX-induced lesions and oxidative damage and boosted the levels of antioxidants in cardiac tissues [69]. AF treatment diminished the immuno-expression of Nuclear factorkappa B (NF-κB) in cardiac tissue and reduced the level of inflammatory cytokines (NO, tumor necrosis factor-α (TNF-α), and interleukin-6 (IL-6)) in the cardiac tissue. The cardioprotective activity of AF against DOX-induced cardiac damage may be attributed to its antioxidant and anti-inflammatory activities [69].

Similarly, Asperimide C (200) and Asperimide D (201) showed a powerful anti-inflammatory effect on NO production in LPS-mediated RAW 264.7 cells, with IC_**50**_ values of 0.78 and 1.26 μM, respectively [85]. The same anti-inflammatory activity was observed for 1,2-dehydro-terredehydroaustin (145) but with IC_**50**_ of 42.3 μM compared to the positive control indomethacin (IC_**50**_ = 30.7 μM) [75].

Versicolactone B (254) had a more potent inhibitory effect than indomethacin against NO production in RAW264.7 mouse macrophages induced by LPS at a concentration of 20 μM [122]. On the other hand, Butyrolactone I (206) and 3′-isoamylene butyrolactone IV (239) showed moderate inhibitory effect (p < 0.05) on NO production with 25.3% and 25.1% inhibition respectively [122]. Astoundingly, metabolites 3′-isoamylene butyrolactone IV (239), Butyrolactone I (206), and Versicolactone B (254) had spectacular inhibitory effects on NO production, with compound (254) being even stronger than indomethacin (50 µM) (a positive control), signifying that (254) could be a potential forefront compound for the advancement of novel anti-inflammatory drugs [122].

In LPS-stimulated RAW264.7 macrophages, Terrusnolides A−D (257–260), demonstrated brilliant inhibitory effects on the production of interleukin-1β (IL-1β) with IC_50_ values of 35.23, 17.89, 16.21, and 21.16 μΜ, respectively, as well as TNF-α with IC_50_ of 42.57, 23.53, 20.45, and 19.83 μM, respectively, and NO with IC_50_ values of 38.15, 21.45, 19.34, and 16.78 μM, respectively, in comparison with indomethacin which had IC_50_ values of 15.67, 21.34, and 18.34 μM, respectively, in the three assays [128]. Terrusnolides A−D could be lead compounds for the development of new anti-inflammatory agents [128]. Likewise, Cowabenzophenone A (286) inhibited the production of IL-6 in LPS-stimulated THP-1 cells with an IC_50_ of 12.1 µg/mL [100].

The highly oxygenated meroterpenoid Terreustoxin C (166) and the sesquiterpenoid Terretonin (133) have been reported to significantly inhibit the proliferation of concanavalin A (Con A)-induced murine T cells at 10 μM [13]. The tryptoquivalines; *N*-dehydroxy tryptoquivaline A (deoxytryptoquivaline) (8) and *O*-deacetyl-tryptoquivaline A (9) exhibited suppression of NF-κB with IC_50_ values of 3.45 and 6.76 μM, respectively, without cytotoxicity, which recommends the capability of interceding a chemopreventive response to cancer [12].

The compounds Terrein (226), Methyl 6-acetyl-4-methoxy-7,8-dihydroxynaphthalene-2-carboxylate (285), and Dihydrogeodin (268) demonstrated potent immunosuppressive activities toward the T cell viability with inhibition rates > 99% at 20 μM, compared to the positive control cyclosporin A [99].

### Antioxidant activity

The butenolides Aspernolide A (210), Aspernolide B (212), Asperteretal E (224), and Butyrolactone III (233) displayed remarkable antioxidant activities in a 2,2-diphenyl-1-picrylhydrazyl (DPPH) radical scavenging assay with IC_50_ of 9.50, 5.89, 6.43, and 10.07 μg/mL, respectively, versus 5.13 μg/mL for ascorbic acid [89].

Similarly, Butyrolactone I (206), 5-[(3,4-dihydro-2,2-dimethyl-2*H*-1-benzopyran-6-yl)-methyl]-3-hydroxy-4-(4-hydroxyphenyl)-2(5*H*)-furanone (209), and Butyrolactone II (232), exhibited strong DPPH radical scavenging capacity with IC_50_ values of 38, 90, and 86 µM, respectively [87]. Butyrolactone I (206) displayed the most potent antioxidant activity indicating that the prenyl and dihydropyran ring moieties could boost the antioxidant activity [87]. Butyrolactone I (206), 5-[(3,4-dihydro-2,2-dimethyl-2*H*-1-benzopyran-6-yl)-methyl]-3-hydroxy-4-(4-hydroxyphenyl)-2(5*H*)-furanone (209), Aspernolide A (210), and Butyrolactone II (232) having a 3-phenyl-4-benzyl substituent, ended up being significantly more active compared to Asperteretal D (223), Asperteretal E (224), Flavipesolide B (227), and Flavipesolide C (228) with 2-benzyl-3-phenyl, which recommended the substituted layout to be a vital factor for the antioxidant activity [87]. Interestingly, Butyrolactone V (235), exhibited a concentration-dependent antioxidant effect with the highest concentration measured 227.0 µM having the greatest impact (_~_95%) similar to that of 567.8 µM of ascorbic acid, however at lower concentration at 22.7 µM, its antioxidant influence (_~_45%,) and was potent than that applied by 56.8 µM of ascorbic acid which was (~ 11%), while Terrein (226) demonstrated intermediate antioxidant activity (_~_44%), at the measured concentrations (64.9, 129.7, 259.5, and 648.7 µM) [102].

Furthermore, 6-(4’-hydroxy-2’-methylphenoxy)-(–)-(3R)-mellein (323) suppressed xanthine oxidase (XO) with an IC_**50**_ value of 243 µM, superior to the standard compounds, 3-*t*-butyl-4-hydroxyanisole (BHA) and propyl gallate (PG), which had IC_50_ values of 591 and 628 μM, respectively [97]. Additionally, compound (323) demonstrated a potent DPPH radical scavenging capacity with an IC_**50**_ value of 159 µM, compared to BHA and PG with IC_50_ values of 44 and 30 μM, respectively [97].

### Anti-Alzheimer’s disease (AD) activity

Alzheimer’s disease (AD) is a progressive cognitive disorder and the most prevalent reason for dementia [79]. It has been found that the β-site amyloid precursor protein-cleaving enzyme (BACE-1) is involved in the abnormal production of the amyloid beta (Aβ), one of the significant histological distinctive features of AD. In addition, acetylcholinesterase (AchE) has been demonstrated to be the most common target for symptomatic improvement in AD because a cholinergic shortage is a constant finding in AD [79]. Hence, the discovery of multitargeted drugs with BACE1 and AchE inhibitory activities plays a substantial role in the treatment of AD [79]. Spiroterreusnoids A–F (150–155) may extend another layout for the development of novel anti-AD drugs [79]. Spiroterreusnoids A–F, with a spiro-dioxolane moiety, represent the first multitargeted natural products [79]. They afford 3,5-DMOA-based meroterpenoid, these compounds are promising BACE-1 inhibitors (IC_**50**_ = 5.86, 25.55, 21.34, 24.98, 27.16, and 25.36 μM, respectively) and moderate AchE inhibitors (IC_**50**_ = 22.18, 27.36, 23.87, 26.85, 32.51, and 31.33 μM, respectively) [79].

It has been reported that some meroterpenoids known as acetylcholinesterase inhibitors can reduce the amount of acetylcholine present in the synapses between cholinergic neurons [42]. Terreulactone A (190), a meroterpenoid containing an unusual fused lactone skeleton*,* suppressed AchE in a dose-dependent pattern with an IC_50_ of 0.2 μM which demonstrated higher activity than a methoxylated derivative of arisugacin B (IC_50_ 0.42 μM) [43]*.* It was likewise found that 16α-hydroxy-5 N-acetylardeemin (32) displayed an inhibitory effect against AchE with an IC_50_ of 58.3 µM (the positive control tacrine IC_50_ was 37.9 μM) [56].

Additionally, Arisugacin A (156), Territrem B (179), Territrem C (180), Territrem D (181), Territrem E (182), and displayed potent AChE inhibitory activity with IC_50_ values of 11.9, 4.2, 20.1, 4.2, and 4.5 nM, respectively [39]. While (156), (179), (180), (181), and (182) were more potent than the positive control Huperzine A (IC50 = 39.3 nM), compound Arisugacin H (158) showed inhibiting AChE activity with an IC_50_ of 5700 nM [39]. The enone group at the A-ring plays a vital role in the AChE inhibition ability of these territrems [39].

In addition, the first DMOA-derived meroterpenoids Asperterpenes E–F and J (136–137 and 141), having cis-fused A/B ring systems, displayed potential BACE1 inhibitory effects with IC_50_ values of 3.3, 5.9, and 31.7 μM, respectively [76]. Asperterpene D-M (135–144), Terretonin D (132), Terretonin (133), and Terretonin G (134) prompted important structure–activity relationship (SAR) interpretations [76]. Aside from compounds (136–137 and 141), all other compounds displayed no apparent inhibitory activities, suggesting that the cis-fused A/B ring system may effectively participate in the BACE-1 inhibitory feature of DMOA-derived meroterpenoids. Additionally, BACE-1 inhibitory activities may be diminished by the open ring D, which was confirmed for compounds (136) and (141) [76]. These SAR investigations will promote further structure improvement of DMOA-derived meroterpenoids for developing novel BACE-1 inhibitors [76]. Besides, compounds Anhydrojavanicin (263), 8-*O*-methylbostrycoidin (63), NGA0187 (98), and Beauvericin (70) exhibited outstanding AChE inhibitory activities with IC_50_ values of 2.01, 6.71, 1.89, and 3.09 μM, respectively, versus 0.003 μM for huperzine A, the positive control [65].

Terreulactones A, B, C, and D (190 -193) suppressed AchE in a dose-dependent manner with IC_**50**_ values of 0.23, 0.09, 0.06, and 0.42 μM, respectively [83]. Terreulactone C (192) had the most powerful AchE inhibitory effect with 3.8, 1.5, 7, and 1.5 times more potent activity than Terreulactones A, B, D, and tacrine, respectively [83]. Terreulactones A-D (190–193), on the other hand, did not inhibit butyrylcholinesterase even at 200 μM [83]. Similarly, Isoterreulactone A (194) suppressed AchE in a dose-dependent manner with an IC_50_ value of 2.5 μM. However, it did not inhibit butyrylcholinesterase even at 500 μM [119]. Isoterreulactone A (194) was 10 times less active than Terreulactone A (190), implying that ring A plays a significant role in AchE inhibitory activity [119].

### Anti-diabetic activity

Type II diabetes (noninsulin-dependent diabetes mellitus), a chronic metabolic disease with impaired glucose metabolism and a series of complications including nephropathy, heart and peripheral vascular complaints, and retinopathy, has become a globally substantial growing public health problem [87]. Treatment of diabetes can be centered around α-glucosidase which plays a significant function in the digestion of disaccharides into monosaccharides, bringing about postprandial hyperglycemia. Hence, α-glucosidase inhibitors can postpone the assimilation of glucose and effectively reduce postprandial hyperglycemia in diabetic patients [87].

The butenolides Butyrolactone I (206), Aspernolide E (214), Butyrolactone VII (240), and displayed notable α-glucosidase inhibitory effects with IC_50_ values of 3.87, 8.06, and 1.37 µM, respectively, being significantly more active than the positive control acarbose (190.2 µM), posing them as potential antidiabetic agents [123].

Conjointly, other butenolide derivatives; (-)-Asperteretal D (223), ( +)-Asperteretal D (223), Asperteretal E (224), Flavipesolide B (227), and Flavipesolide C (228) showed promising activity with IC_50_ values of 9.98, 8.65, 13.36, 10.3, and 7.63 μM, respectively [87]. The enantiomers (-)-223 and ( +)-223 showed almost the same α-glucosidase inhibitory activities, hence the distinction of chirality had an insignificant effect on the activity [87].

In addition, 5-[(3,4-dihydro-2,2-dimethyl-2*H*-1-benzopyran-6-yl)-methyl]-3-hydroxy-4-(4-hydroxyphenyl)-2(5*H*)-furanone (209) and Aspernolide A (210) inhibited α-glucosidase with IC_50_ values of 11.65 and 47.33 μM, respectively, compared to the positive control acarbose (IC_50_ 320 μM) [87]. Comparing the IC_50_ values of (209) and (210) suggests that the methoxycarbonyl group at C-4 in (210) negatively affects the function of α-glucosidase inhibitory activity [87]. Furthermore, a hydroxyl group at C-2ʺʹ in Butyrolactone V (235), significantly reduced the activity compared to Aspernolide A (210) [87]. In contrast to Butyrolactone II (232), Butyrolactone I (206) demonstrated greater activity, confirming that the prenyl chain makes a crucial contribution to the α-glucosidase inhibitory effect [87].

On the other hand, Amauromine B (43), a prenylated diketopiperazine alkaloid, and the meroterpenoid Austalide N (176) displayed more powerful α-glucosidase inhibitory activities than the positive control acarbose with IC_50_ values of 0.30, 0.40 and 0.66 mM, respectively [110]. The butenolide Versicolactone G (255) exhibited powerful α-glucosidase inhibitory activity with an IC_50_ value of 104.8 μM, versus 154.7 µM for acarbose [45]. It has been reported that Cowabenzophenone A (286) demonstrated an α-glucosidase inhibitory activity with an IC_50_ value of 7.8 μM [100]. In addition, the Kodaistatins A–D (297–300) suppress the transport protein glucose-6-phosphate T1 translocase in the nanomolar range (IC_50_ = 80–130 nM). This protein is implicated in transporting glucose-6-phosphate from the cytoplasm into the endoplasmatic reticulum of hepatocytes, the site of the final step of both gluconeogenesis and glycogenolysis [93]. Reportedly, Kodaistatin A (297) suppressed glucose-6-phosphatase activity in untreated rat liver microsomes with IC_**50**_ of 0.08 μM, while Kodaistatin C (299) IC_**50**_ was 0.13 μM, and, conversely, the pyrophosphatase activity for both Kodaistatin A and C of untreated microsomes stayed uninfluenced, and disruption of the microsomal membranes totally revoked the inhibition of glucose-6-phosphatase activity shown in untreated microsomes, (The term "untreated" refers to microsomal vesicles that have been prepared and used without further treatment) [94]. Moreover, Terrelumamide A and B (84–85) improve insulin sensitivity by enhancing the production of adiponectin in the hBM-MSCs adipogenesis model [52]. Glibenclamide and aspirin were utilized as double positive controls as their pharmacological mechanisms for improving insulin sensitivity are divergent [52]. Whilst the EC_**50**_ values for glibenclamide and aspirin were 3.47 and 145.6 mM, respectively, compounds (84) and (85) had EC_**50**_ of 37.1 and 91.9 mM, respectively [52].

### Anti-β-glucuronidase activity

The hydrolysis of glucuronides is catalyzed by the acid hydrolase *β*-Glucuronidase to produce their respective aglycones and free glucuronic acid [88]. Nevertheless, colon cancer is associated with the over-expression of this enzyme in intestinal bacteria in humans and rats [88]. Moreover, gallstone formation is linked to *β*-glucuronidase of bacteria existing in the biliary tract [88]. Consequently, in the treatment of related diseases, particular inhibitors of *β*-glucuronidase could be developed [88]. The butyrolactone 3-hydroxy-4-(4-hydroxyphenyl)-5-methoxycarbonyl-5-(4-hydroxy-3-formylbenzyl) -2,5-dihydro-2-furanone (229) exhibited substantial *β*-Glucuronidase enzyme inhibition activity, with an IC_**50**_ value of 6.2 μM, however, Butyrolactone I (206) and ( +)-Asterrelenin (14) displayed moderate *β*-Glucuronidase inhibitory activity with IC_**50**_ values of 96.7 and 126 μM, respectively, whereas the positive control, glucosaccharo-(1,4)-lactone had IC_**50**_ = 48.4 μM [88]. The noteworthy inhibitory activity of the compound (229) is most likely because of the possible proton acceptance from the carboxylic acid at the active site of the enzyme [88].

### Anti-tumor and cytotoxicity activity

The in vitro cytotoxicity assay against the human nasopharyngeal epidermoid carcinoma (KB) cell line for 10-phenyl-[11]-cytochalasin Z17 (39) showed moderate cytotoxicity with an IC_**50**_ value of 26.2 µM, (doxorubicin IC_**50**_ was 0.01 µM) [55]. An adequate antitumor activity has been provided by Butyrolactone I (206) and Butyrolactone V (235) against the breast cancer cell lines MDA-MB-231 and MCF-7 with IC_50_ values less than tamoxifen (IC_50_ = 61.6 and 53.0 µM, respectively) [102]. Compound (206) exerts a cytotoxic effect on human promyelocytic leukemia cells (IC_**50**_ of 18.85 µM) [61, 102].

A selective antiproliferative effect against prostate (PC-3) and kidney (786–0) cancer cell lines (IC_50_ of 22.93 and 48.55 μM, respectively) was reported by the prenylated indole alkaloid, Giluterrin (1) among six different tested cancer cell lines; U251 (glioma), MCF7 (breast), 786–0 (kidney), NCI-H460 (nonsmall cell lung cancer), PC-3 (prostate), HT-29 (colon) [44].

Terrein (226) demonstrated potent cytotoxicity against breast cancer MCF-7 cells [43] through induction of apoptosis via activating the caspase-7 pathway and restraining the Akt signaling pathway, Moreover, terrein is a powerful inhibitor of the 20S proteasome and can inhibit keratinocyte proliferation and melanogenesis [43].

Compounds Dehydrocurvularin (340) and 11-methoxycurvularin (341) demonstrated notable cytotoxicity against a panel of four cancer cell lines: human non-small cell lung carcinoma (NCIH460), human breast carcinoma (MCF-7), human glioma (SF268), human pancreatic cancer (MIA Pa Ca-2), with IC_**50**_ values 1.1, 1.3, 2.5, 1.9 µM, and 0.9, 0.6, 0.9, 1.2 µM, respectively, and against the normal human primary fibroblast (WI-38) cells, (340) and (341) had IC_50_ of 3.6 µM and 1.7 µM, respectively [49]. Terrequinone A (22) and 11- hydroxycurvularin (339), on the other hand, were moderately active with IC_**50**_ values of 5.6, 6.8, 13.9, 5.4 µM and 2.1, 2, 4.1, 3.3 µM, respectively, and compound (339) had an IC_**50**_ of 11.6 µM against the normal human primary fibroblast (WI-38) cells [49].

Moreover, the cytotoxic activities of Asperterzine (54), Bisdethiobis(methylthio)-acetylapoaranotin (55), Bisdethiodi(methylthio)-acetylaranotin (56), were assessed against HL-60 (human promyelocytic leukemia cells) cell line [60]. Compounds (55) and (56) displayed powerful inhibitory impacts with IC_50_ values of 16.30 and 9.34 μmol/L [60]. Asperterreusine A (314) demonstrated cytotoxicity against human cancer cell lines HL-60 and SW-480cell lines with IC_**50**_ values of 15.3 and 25.7 μM, respectively [72]. Aspernolide A (210) displayed moderated cytotoxicity towards HL-60 (human promyelocytic leukemia cells) with IC_**50**_ = 39.36 μM, whereas the positive control was 5-Fluorouracil (5-FU) with IC_50_ value of 2.80 μM [61]. Botryosphaerin F (115) demonstrated a significant inhibitory effect against both human breast cancer (MCF-7) and human promyelocytic leukemia (HL-60) cells with 50% inhibition of cell growth (IC_50_ = 4.49 and 3.43 μM, respectively), and 13,14,15,16-tetranorlabd-7-ene-19,6b:12,17-diolide (116) showed potent activity against human breast cancer cell MCF-7 cell with IC_50_ values of 2.79 μM [73].

Moreover, it has been found that compounds 3*β*,5*α*-dihydroxy-(22*E*,24*R*)-ergosta-7,22-dien-6-one (96) and Beauvericin (70) showed potent or moderate cytotoxic effects towards human breast cancer cells (MCF-7), lung cancer cells (A549), cervix carcinoma cells (Hela) and human nasopharyngeal carcinoma cells (KB) with IC_50_ values 4.98 and 2.02 (MCF-7), 1.95 and 0.82 (A549), 0.68 and 1.14 (Hela), and 1.50 and 1.10 μM (KB), respectively, while compound 3β,5α,14α-trihydroxy-(22E,24R)-ergosta-7,22-dien-6-one (97) possessed poor inhibitory activities towards these tumor cell lines, however epirubicin, the positive control presented IC_50_ values 1.07 (MCF-7),0.79 (A549), 0.42 (Hela), 0.05 (KB) [65]. In addition to, Asperterrestide A (71), a cytotoxic cyclic tetrapeptide manifested cytotoxicity against human leukemic monocyte lymphoma U937and acute lymphoblastic leukemia MOLT-4 cell lines with IC_50_ values of 6.4 and 6.2 μM, respectively and taxol was utilized as a positive control against the U937, and MOLT-4 cell lines with IC_50_ values 1.9 and 1.8 μM, respectively [40].

Reportedly, compounds ( ±)-Asperteretone F (261a and 261b) displayed potential cytotoxic activities against three human pancreatic cancer cell lines, including AsPC-1, SW1990, and PANC-1 cells, with IC_**50**_ values of 9.5 μM, 11.7 μM, 9.8 μM and 9.9 μM, 10.3 μM, 15.6 μM, respectively, hence these research results may give a premise to the development of butenolides as the genesis of promising agents for pancreatic cancer [92].

Cowabenzophenone A (286) demonstrated a cell viability value of 32%, coming about the cytotoxicity to be 68%, additionally exhibited cytotoxicity against HCT 116 colon cancer cell line with IC_50_ values of 10.1 μM, whereas doxorubicin was used as a positive control with an IC_**50**_ value of 9.74 μM [100].

Terrstatins A and B (294 and 295) were evaluated for their cytotoxicity against five human tumor cell lines, including the HeLa, SW480, AsPC-1, SW1990, and PANC-1 cell lines, and were shown to be inactive (IC_50_ > 40 μM) [92].

### Anti-hyperlipidemic and anti-atherosclerotic activity

One of the greatest breakthroughs in industrial microbiology is the isolation of the natural statins, compactin, mevastatin, and lovastatin (mevinolin) (290) from *A. terreus* [129], in addition to the semi-synthetic derivative simvastatin [137]. Statins are a class of drugs that inhibit HMG-CoA reductase, resulting in reduced cholesterol production [129]. In the 1970s, the statins were first discovered by Dr. Akira Endo, the Japanese microbiologist, in the filamentous fungi *Penicillium (P.) citrinum* and later in *A. terreus*; lovastatin was first discovered in 1978 by Alberts, Chen, and others, and for a long time, fungi were the only source for the statins [129, 138]. The United States Food and Drug Administration approved the first statin, lovastatin, as anti-hypercholesterolemic drug in August 1987 [129]. The structure of chemically synthesized statins, such as atorvastatin, rosuvastatin, fluvastatin, and cerivastatin, differs from natural statins; nonetheless, there is a similitude to natural statins in the HMG CoA-like inhibitory moiety [129]. Lovastatin (290) has long been used to lower cholesterol and lipid levels in several diseases, it is well-known for decreasing cholesterol and increasing the hepatic uptake of LDL-C via upregulating low-density lipoprotein receptors (LDLR) and because it has a structure similar to HMG-CoA, it can bind competitively to HMG-CoA reductase (HMGR) and act as a hypolipidemic medication [138]. Leach et al. reported that a Lipoprotein-associated phospholipase A2 (Lp-PLA2) inhibitor decreased the growth of atherosclerotic plaque in the Watanabe heritable hyperlipidemic (WHHL) rabbit study. While *R* (–)-glycerol-monolinoleate (335) showed moderate Lp-PLA2 inhibitory activity with IC_50_ value of 45.0 µM methyl linoleate had no effect on Lp-PLA2. [139].

### UV-a protecting activity

Terreusinone (26), holding a dipyrroloquinone moiety, demonstrated an ultraviolet-A (UV-A) absorbing activity with an ED_50_ value of 70 μg/mL, which is stronger than oxybenzone currently in use as a sunscreen [54].

## Conclusions and future perspectives

The main goal of this in-depth literature review is to bring together the burgeoning significance of biochemical assessment of endophytic fungi, with a particular focus on up-to-date chemical and pharmacological information on *A. terreus* derived from sundry sources, which could significantly advance the ongoing innovation and development of novel therapeutic agents. The systematic review sheds light on the secondary metabolites discovered almost from the year 1987 to the first quarter of the year 2022 after being isolated from the endophytic fungus *A. terreus* living inside the internal tissues of various terrestrial and marine sources from diverse geographical origins. Based on the presented data, *A. terreus* is a fructiferous source of secondary metabolites with a wide range of chemical scaffolds and biological activities. The reported metabolites originated from a diverse range of chemical classes involving indole alkaloids, ardeemins, cytochalasins, diketopiperazines, epipolythiodiketopiperazines, peptides, triterpenes, sesquiterpenes, sesterterpenes, meroterpenoids, butenolides and butyrolactones, statins, isocoumarins, and benzophenones. However, γ-butyrolactones and meroterpenoids constitute major classes of secondary metabolites produced by *A. terreus*. The isolated compounds revealed a striking divergence of biological impacts that enticed much consideration, demonstrating acetylcholinesterase (AChE) inhibitory, anti-tuberculosis, antibacterial, antifungal, antifouling, antiviral, antileishmanial, antischistosomal, antifilarial, antiplasmodial, anti-inflammatory, antioxidant, anti-diabetic, and mosquitocidal activities. Of the 346 compounds that have been identified and reported, 172 of them have been shown to have biological activities. Further biological studies on these natural products need to be done since almost half of the isolated compounds (~ 50%) were either inactive or not biologically investigated in the assays that were conducted, resulting in a broad undiscovered area. The abundance of published data unequivocally attests to the pharmacological activities of *A. terreus*. However, the clinical benefits of *A. terreus* are yet unknown, despite years of extensive investigation. It will be challenging for the therapeutic natural secondary metabolites isolated from *A. terreus* to approach allopathic mainstream medicine as long as randomized, placebo-controlled clinical studies are not provided. This is a significant drawback of many other naturally occurring compounds, not just those isolated from *A. terreus*. The ability to prove therapeutic action in a clinical context is a crucial task for integrative medicine research. For the benefit of patients all across the world, it is hoped in the not-too-distant future that this major challenge will be resolved. Moreover, the application of recent technologies to explore the biosynthesis of the promising metabolites at the molecular level provides an open area for research including the application of epigenetic modifiers and OSMAC approach to maximize the benefit from this outstanding source of secondary metabolites.

## Data Availability

Not applicable.
